# The Southern European Atlantic diet and all-cause and cause-specific mortality: a European multicohort study

**DOI:** 10.1093/eurjpc/zwad370

**Published:** 2023-12-15

**Authors:** Adrián Carballo-Casla, Denes Stefler, Rosario Ortolá, Yuntao Chen, Anika Knuppel, Ruzena Kubinova, Andrzej Pajak, Fernando Rodríguez-Artalejo, Eric J Brunner, Martin Bobak

**Affiliations:** Department of Preventive Medicine and Public Health, Universidad Autónoma de Madrid, Calle del Arzobispo Morcillo 4, 28029 Madrid, Spain; Center for Networked Biomedical Research in Epidemiology and Public Health (CIBERESP), Avenida de Monforte de Lemos 3-5, 28029 Madrid, Spain; Department of Epidemiology and Public Health, University College London, 1-19 Torrington Place, London WC1E 7HB, UK; Aging Research Center, Department of Neurobiology, Care Sciences and Society, Karolinska Institutet & Stockholm University, Tomtebodavägen 18 A SE-171 77 Stockholm, Sweden; Department of Epidemiology and Public Health, University College London, 1-19 Torrington Place, London WC1E 7HB, UK; Department of Preventive Medicine and Public Health, Universidad Autónoma de Madrid, Calle del Arzobispo Morcillo 4, 28029 Madrid, Spain; Center for Networked Biomedical Research in Epidemiology and Public Health (CIBERESP), Avenida de Monforte de Lemos 3-5, 28029 Madrid, Spain; Department of Epidemiology and Public Health, University College London, 1-19 Torrington Place, London WC1E 7HB, UK; Independent researcher; Department of Environmental Health and Population Health Monitoring, National Institute of Public Health, Šrobárova 49/48, 100 00 Prague, Czech Republic; Department of Epidemiology and Population Studies, Faculty of Health Sciences, Institute of Public Health, Jagiellonian University Medical College, Ulica Skawińska 8, 31-066 Krakow, Poland; Department of Preventive Medicine and Public Health, Universidad Autónoma de Madrid, Calle del Arzobispo Morcillo 4, 28029 Madrid, Spain; Center for Networked Biomedical Research in Epidemiology and Public Health (CIBERESP), Avenida de Monforte de Lemos 3-5, 28029 Madrid, Spain; Cardiovascular and Nutritional Epidemiology Group, CEI UAM+CSIC, IMDEA Research Institute on Food & Health Sciences, Carretera de Canto Blanco 8, 28049 Madrid, Spain; Department of Epidemiology and Public Health, University College London, 1-19 Torrington Place, London WC1E 7HB, UK; Department of Epidemiology and Public Health, University College London, 1-19 Torrington Place, London WC1E 7HB, UK; RECETOX, Faculty of Science, Masaryk University, Kotlářská 267/2, 611 37 Brno, Czech Republic

**Keywords:** Mediterranean diet, Seafood, Processed meat, Alcohol, Longitudinal, Coronary heart disease, Stroke, Death, Cox model, Public health

## Abstract

**Aims:**

The Southern European Atlantic diet (SEAD) is the traditional dietary pattern of northwestern Spain and northern Portugal, but it may resemble that of central, eastern, and western European countries. The SEAD has been found associated with lower risk of myocardial infarction and mortality in older adults, but it is uncertain whether this association also exists in other European populations and if it is similar as that found in its countries of origin.

**Methods and results:**

We conducted a prospective analysis of four cohorts with 35 917 subjects aged 18–96 years: ENRICA (Spain), HAPIEE (Czechia and Poland), and Whitehall II (United Kingdom). The SEAD comprised fresh fish, cod, red meat and pork products, dairy, legumes and vegetables, vegetable soup, potatoes, whole-grain bread, and moderate wine consumption. Associations were adjusted for sociodemographic variables, energy intake, lifestyle, and morbidity. After a median follow-up of 13.6 years (range = 0–15), we recorded 4 973 all-cause, 1 581 cardiovascular, and 1 814 cancer deaths. Higher adherence to the SEAD was associated with lower mortality in the pooled sample. Fully adjusted hazard ratios and 95% confidence interval per 1-standard deviation increment in the SEAD were 0.92 (0.89, 0.95), 0.91 (0.86, 0.96), and 0.94 (0.89, 0.99) for all-cause, cardiovascular, and cancer mortality, respectively. The association of the SEAD with all-cause mortality was not significantly different between countries [Spain = 0.93 (0.88, 0.99), Czechia = 0.94 (0.89,0.99), Poland = 0.89 (0.85, 0.93), United Kingdom = 0.98 (0.89, 1.07); *P* for interaction = 0.16].

**Conclusion:**

The SEAD was associated with lower all-cause, cardiovascular, and cancer mortality in southern, central, eastern, and western European populations. Associations were of similar magnitude as those found for existing healthy dietary patterns.

**See the editorial comment for this article ‘The diet lumberjacks needed’, by I. Anttila** ***et al*** **., https://doi.org/10.1093/eurjpc/zwae024.**

## Introduction

The Southern European Atlantic diet (SEAD) is the traditional dietary pattern of northwestern Spain and northern Portugal. In these regions, staple foods were fish, particularly cod; red meat and pork products; dairy; legumes; vegetables and potatoes, often eaten in soups; whole-grain bread; and wine.^1–4^ Conversely, the consumption of olive oil, fresh fruits, and nuts was not widespread until the second half of the 20th century.^1–4^ Though some of these characteristics are distinctive to the SEAD, this traditional diet may still resemble that of Nordic, central, eastern, and western European countries.^5,6^

Increased adherence to the SEAD has been associated with healthier gut microbiota^7^ and reduced levels of several cardiovascular risk factors, such as C-reactive protein, triglycerides, insulin, insulin resistance, pulse wave velocity, systolic blood pressure, total cholesterol, body mass index (BMI), and waist circumference.^8–13^ Reductions in the latter three risk factors have been reproduced in a randomized controlled trial.^14^ Finally, increased adherence to the SEAD has also been linked to decreased risk of myocardial infarction and all-cause mortality in older adults.^4,15^

However, available studies on the SEAD have only been conducted in its countries of origin. Since Spain and Portugal have lower mortality rates from cardiovascular disease and higher life expectancy than other European countries,^4,5^ the external validity of previous findings is unclear. In addition, the consumption of some of the SEAD food groups might be inconsistent with healthy diet recommendations (i.e. red meat and pork products seem to be associated with higher risk of cardiometabolic disease, cancer, and all-cause mortality,^16–18^ potatoes may increase the risk of type 2 diabetes,^19^ and there might be no mortality benefits from moderate alcohol intake).^20,21^ These food groups of the SEAD diverge as well from those of existing evidence-based dietary patterns, like the Alternate Healthy Eating Index (AHEI) or the Dietary Approaches to Stop Hypertension (DASH).^22,23^

To assess the generalizability of previous studies on the SEAD to other European countries and younger populations, as well as its impact on cardiovascular disease and cancer, we used cohort data from four European countries (Spain, Czechia, Poland, and the United Kingdom) to examine the association between adherence to the SEAD and all-cause and cause-specific mortality.

## Methods

### Study design and participants

The cohorts included in the analyses are briefly described below. The ENRICA is a study representative of the non-institutionalized population aged ≥18 years in Spain. Study participants were recruited between 2 008 and 2 010. Data on sociodemographic variables, lifestyle, and morbidity were gathered through telephone interviews, whereas a detailed diet history, a comprehensive set of physical measurements, and a blood test were collected at home visits by trained personnel. The Clinical Research Ethics Committee of the ‘La Paz’ University Hospital in Madrid approved the research protocol, and all subjects gave written informed consent.^24^

The HAPIEE cohort was set up in 2 002–2 005 and recruited random samples of men and women aged 45–69 years from six cities in Czechia and Krakow (Poland). It included an interview that gathered data on health, lifestyle, diet via a food frequency questionnaire (FFQ), and socioeconomic circumstances. A short examination, including physical measurements and a blood test, was also conducted. The study was approved by the ethics committee at University College London and by the ethics committee in each participating centre. All participants gave written informed consent.^25^

The Whitehall II is a cohort study of civil servants from 20 civil service departments in London (United Kingdom). Participants undergo medical examinations and fill out an FFQ every 5 years, and they complete an array of questionnaires in and between these screening phases.^26–28^ We used data from the fifth phase of the study, which took place between 1 997 and 1 999, because its participants were aged 45–69 years, same as HAPIEE’s. The University College London Ethics Committee approved the study. After the subjects were given a complete description of the study, written informed consent was obtained from all participants.^29^

### Study variables

#### Diet

In Spain, food consumption was obtained with a validated electronic diet history. Subjects could report up to 861 foods and recipes habitually consumed in the country. Portion sizes were estimated with the help of 127 digitized photographs and household measures. Nutrient and energy intake were derived from Spanish and other standard food composition tables.^30^ In Czechia, Poland, and the United Kingdom, dietary data were collected with a semi-quantitative FFQ that consisted of 136, 147, and 116 foods and beverages, respectively—note that the FFQ used in HAPIEE was an expanded version of that used in Whitehall II. In these questionnaires, participants indicated how frequently they consumed foods and drinks by using a nine-point scale, ranging from ‘never or less than once a month’ to ‘more than 6 times a day’. The McCance and Widdowson’s food composition tables were used to estimate nutrient and energy intake.^26,31^

To estimate the adherence to the SEAD, we used a scoring method proposed by Oliveira *et al.*^4^ which includes the nine food groups that have been considered by most studies on this dietary pattern:^8–12,15^ fresh fish, excluding cod; cod; red meat and pork products; dairy; legumes and vegetables, excluding those consumed in soup; vegetable soup, potatoes, regardless of the cooking method; whole-grain bread; and wine. The first eight food groups were scored 1–3 points each: 1 point was given to the subjects consuming <1 serving/week, 2 points to those consuming 1 to 7 servings/week, and 3 points to those consuming ≥1 serving/day. Wine consumption was scored 0–1 points: men who drank >0 and ≤2 glasses/day and women who drank >0 and ≤1 glasses/day were given 1 point, whereas no points were given for >2 glasses/day in men, >1 glass/day in women, or 0 glasses/day. The adherence to the SEAD was computed as the sum of these 9 scores; it ranged from 8 to 25, with higher values indicating better adherence.

To put the SEAD in context, we compared the study associations with those of two frequently used, evidence-based, healthy dietary patterns: the AHEI, whose food groups were based on its association with chronic disease risk,^22^ and the DASH, which has shown to lower blood pressure and cardiovascular disease risk.^23^

#### Mortality

In Spain, vital status was ascertained with the National Death Index, an information system that collects mortality data from civil registries nationwide. Information on causes of death was based on the death certificates of Spanish residents, taken from the National Institute of Statistics of Spain. Study participants were followed until December 2 020 to ascertain causes of death and until January 2 022 for vital status.^15^

The national death register in Czechia and local and regional death registers in Poland were used to track deaths in these countries. In Czechia and Poland, individuals were followed up for mortality until June 2 021 and August 2 017, respectively.^6^

In the United Kingdom, follow-up for mortality through the National Health Services Central Registry provided the date and cause of death. The identification number assigned to all British citizens was used for linkage to the National Health Services death and electronic patient records. Mortality data were available until February 2 021.^29^

Causes of death were classified and grouped according to the International Classification of Diseases (ICD), tenth revision (Spain, Czechia, and Poland), or according to the ninth or tenth revision (United Kingdom). We considered ICD-10 codes ranging from I00 to I99 (ICD-9 from 390 to 459) to be cardiovascular deaths and ICD-10 codes from C00 to D48 (ICD-9 from 140 to 239) to be cancer deaths. We also analysed three of the leading causes of cardiovascular death (ischaemic heart diseases, cerebrovascular diseases, and heart failure) and cancer death (digestive organs, respiratory and intrathoracic organs, and lymphoid, haematopoietic, and related tissue).

#### Covariates

We used baseline data on several potential confounders of the association between the SEAD and mortality: first, sociodemographic variables, specifically age, sex, educational level (primary or less, secondary or vocational, university, or no data), and marital status (single, married/cohabiting, divorced/separated or widowed, or no data); second, energy intake (kcal/day); third, lifestyle: tobacco smoking (never, former, current, or no data) and leisure time physical activity (hours/week);^24,25,29^ and fourth, morbidity. We considered that subjects had diabetes if they either had blood glucose levels ≥7 mmol/L, were being treated with antidiabetic drugs, or reported a diabetes diagnosis. The medical diagnoses of cardiovascular disease (coronary heart disease, stroke, or heart failure), chronic lung disease, musculoskeletal disease (osteoarthritis, arthritis, or hip fracture), and cancer were self-reported in Spain, Czechia, and Poland, while they were verified through primary care and hospital records in the United Kingdom. Depression was defined as self-reported diagnosis or use of prescribed antidepressant medication in Spain, a score ≥20 on the Center for Epidemiological Studies Depression scale in Czechia and Poland, and a score ≥4 on a depression subscale from the General Health Questionnaire or use of prescribed antidepressant medication in the United Kingdom.^24,25,29,32^

In sensitivity analyses, we used baseline data on systolic and diastolic blood pressure (i.e. average between ≥2 measurements taken during the home visits or clinical examinations) and antihypertensive drug use (self-reported by the study participants). We defined hypertension as systolic blood pressure ≥130 mm of mercury, diastolic blood pressure ≥80 mm of mercury, or use of antihypertensive medication. Measured weight and height were used to calculate BMI (categorized as <25, 25–30, ≥ 30 kg/m^2^, or no data).^24,25,29^

### Statistical analyses

#### Analytical sample

Of the 40 560 subjects recruited at baseline (13 105 from ENRICA, 19 585 from HAPIEE, and 7 870 from phase 5 of Whitehall II), we excluded 4 643 subjects with inadequate data (1 091 had no information on mortality and 3 578 on diet; note that some subjects lacked data in both variables). Hence, the pooled analytical sample for all-cause mortality comprised 35 917 individuals. Since 450 of them did not have data on causes of death, the pooled analytical sample for cause-specific mortality comprised 35 467 individuals (see Supplementary material online, *Figure S1*).

#### Main statistical analyses

The association between the SEAD and mortality was summarized with hazard ratios (HR) and their 95% confidence interval (CI), estimated with Cox proportional hazards regression. We used age as the time scale, so that subjects entered the risk set at their baseline age and exited it at their event/censoring age. Analyses conducted in the pooled sample assumed equal HR across countries but a baseline hazard unique to each country (stratified estimation), whereas country-level estimates were obtained from models with interaction terms (defined as the product of the SEAD by each country), using the *lincom* command in Stata® (StataCorp LLC), version 17.0. To control for potential confounding, two incrementally adjusted models were used: (i) adjusted for sociodemographic characteristics and (ii) additionally adjusted for energy intake, lifestyle, and morbidity. To maximize the comparability of results across countries, participants were censored at 15 years of follow-up.

The country-specific adherence to the SEAD was modelled in the analyses as (i) a continuous variable [per 1-standard deviation (SD) increment]; (ii) quartiles, using the lowest one as reference; and (iii) a restricted cubic spline (see Supplementary material online, *Appendix S1*). The adherence to the AHEI and the DASH was modelled alike.

#### Sensitivity analyses and interactions

We conducted several sensitivity analyses: First, we examined the association of each of the SEAD food groups with mortality. To assure that the associations were independent of the consumption of other foods, these analyses were additionally adjusted for all other SEAD food groups. Second, we calculated an alternate version of the SEAD, optimized for potential public health interventions. Here, we reversely scored the consumption of red meat/pork products and potatoes and did not score wine consumption. To avoid potential confounding, these analyses were additionally adjusted for wine consumption. Third, since beer and other alcoholic beverages may be consumed more often than wine in some central, eastern, and western European countries, we computed the SEAD considering total alcohol intake instead of wine consumption. Specifically, men who had >0 and ≤20 g/day of alcohol and women who had >0 and ≤10 g/day were given 1 point, whereas no points were given for >20 g/day in men, > 10 g/day in women, or 0 g/day. Fourth, to check for possible dietary confounding of the study associations, we adjusted the analyses for the consumption of common foods not included in the SEAD (fruits, nuts, and sugar-sweetened beverages). Fifth and sixth, we alternatively adjusted the models for hypertension and BMI, as both variables could impact dietary choices and mortality. Seventh, since morbidity may mediate—rather than confound—the study associations, we did not adjust the analyses for chronic diseases at baseline. Eighth, to minimize the potential for reverse causation—health status influencing food consumption, rather than the opposite—we alternatively omitted the first year of follow-up and excluded the subjects with severe chronic diseases at baseline (diabetes, cardiovascular disease history, chronic lung disease, or cancer history). Lastly, HR may not be constant along the follow-up and their significance depends on the baseline hazard. To provide an alternate measure of the association between the SEAD and mortality, we computed pseudo-observations for the survival function, which were entered in a generalized linear model to estimate relative risks of death at 15 years, using the *stpsurv* command in Stata®.

We also examined if sociodemographic variables, lifestyle, and morbidity modified the main study associations by using likelihood ratio tests that compared models with and without interaction terms, defined as the product of the SEAD by said variables.

## Results

### Description of study participants

Compared with individuals in the lowest quartile of the SEAD (see Supplementary material online, *Appendix S1*), those in the higher quartiles were older, more often men, married or cohabiting and had a higher educational level. Their energy intake was higher as well, though they were less likely to smoke and did more physical activity. Finally, their prevalence of diabetes, cardiovascular disease, musculoskeletal disease, and chronic lung disease was comparatively high, while they suffered less from depression (*Table 1*). The distribution of characteristics of study participants by country is shown in Supplementary material online, *Table S1*.

**Table 1:** Characteristics of the pooled sample, by quartiles of the Southern European Atlantic Diet

N	Southern European Atlantic Diet				
	Quartile 1 (lowest)	Quartile 2	Quartile 3	Quartile 4 (highest)	Total
	9 981	8 065	8 161	9 710	35 917
Sex—male (%)	4 733 (47.4)	3 987 (49.4)	4 328 (53.0)	5 325 (54.8)*	18 373 (51.2)
Age (years)	53.5 (13.3)	53.3 (12.9)	54.3 (11.7)	55.8 (10.1)*	54.2 (12.1)
Educational level (%)					
Primary or less	1 815 (18.2)	1 399 (17.3)	1 249 (15.3)	1 215 (12.5)*	5 678 (15.8)
Secondary or vocational	5 796 (58.1)	4 567 (56.6)	4 705 (57.7)	5 576 (57.4)	20 644 (57.5)
University	2 311 (23.2)	2 052 (25.4)	2 172 (26.6)	2 890 (29.8)	9 425 (26.2)
No data	59 (0.59)	47 (0.58)	35 (0.43)	29 (0.30)	170 (0.47)
Marital status (%)					
Single	1 561 (15.6)	1 125 (13.9)	935 (11.5)	803 (8.27)*	4 424 (12.3)
Married/cohabiting	6 517 (65.3)	5 639 (69.9)	6 042 (74.0)	7 552 (77.8)	25 750 (71.7)
Divorced/separated or widowed	1 731 (17.3)	1 195 (14.8)	1 086 (13.3)	1 261 (13.0)	5 273 (14.7)
No data	172 (1.72)	106 (1.31)	98 (1.20)	94 (0.97)	470 (1.31)
Tobacco smoking (%)					
Never	4 351 (43.6)	3 543 (43.9)	3 742 (45.9)	4 373 (45.0)*	16 009 (44.6)
Former	2 760 (27.7)	2 333 (28.9)	2 419 (29.6)	3 043 (31.3)	10 555 (29.4)
Current	2 780 (27.9)	2 146 (26.6)	1 959 (24.0)	2 249 (23.2)	9 134 (25.4)
No data	90 (0.90)	43 (0.53)	41 (0.50)	45 (0.46)	219 (0.61)
Physical activity (hours/week)	19.1 (14.7)	20.2 (14.6)	19.7 (14.5)	20.0 (14.5)*	19.7 (14.6)
Energy intake (kcal/day)	1 811 (598)	2 082 (628)	2 238 (650)	2 464 (737)*	2 145 (701)
Diabetes (%)	920 (9.22)	825 (10.2)	882 (10.8)	1 169 (12.0)*	3 796 (10.6)
Cardiovascular disease history (%)	1 218 (12.2)	892 (11.1)	957 (11.7)	1 383 (14.2)*	4 450 (12.4)
Musculoskeletal disease (%)	4 226 (42.3)	3 327 (41.3)	3 662 (44.9)	4 769 (49.1)*	15 984 (44.5)
Chronic lung disease (%)	1 149 (11.5)	839 (10.4)	907 (11.1)	1 150 (11.8)*	4 045 (11.3)
Cancer history (%)	370 (3.71)	293 (3.63)	292 (3.58)	390 (4.02)	1 345 (3.74)
Depression (%)	1 405 (14.1)	921 (11.4)	867 (10.6)	1 033 (10.6)*	4 226 (11.8)

Values are numbers (%) or means (standard deviations).

^*^ *P*-value < 0.05 for differences in means (ANOVA) or proportions (Pearson’s χ^2^) across quartiles of the Southern European Atlantic Diet.

Regarding the consumption of the SEAD food groups, most participants ate cod and whole-grain bread less often than once a week; fresh fish, red meat/pork products, vegetable soup, and potatoes 1–6 times/week; and dairy and legumes/vegetables daily. The majority of participants did not drink wine or had >2 glasses/day in men and >1 glass/day in women (see Supplementary material online, *Table S2*).

### Main results

**Figure 1:**
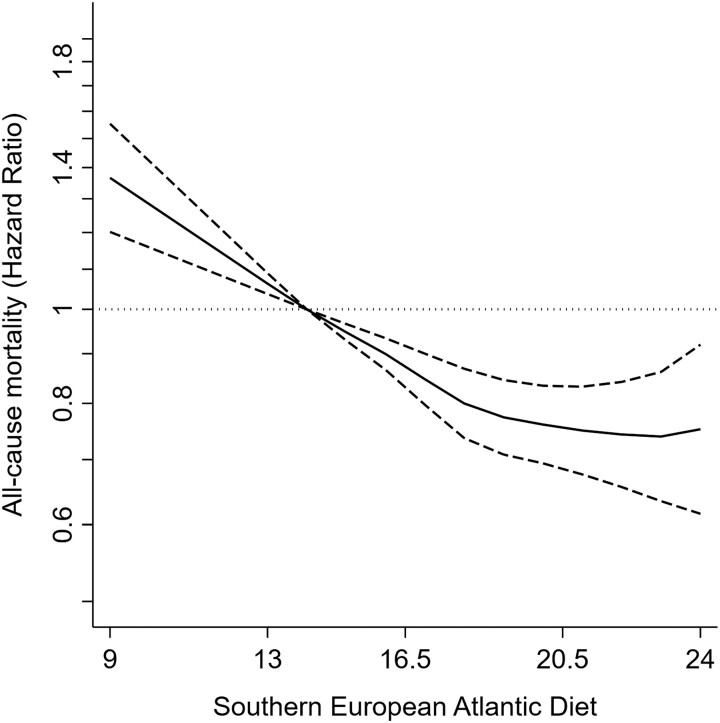
Hazard ratios (95% confidence interval) for the association between the Southern European Atlantic Diet and 13.6-year all-cause mortality in the pooled sample. Cox regression model stratified for country and adjusted as Model 2 in *Table 2*: sex, age, educational level (primary or less, secondary or vocational, university, or no data), marital status (single, married/cohabiting, divorced/separated or widowed, or no data), tobacco smoking (never, former, current, or no data), leisure time physical activity (hours/week), energy intake (kcal/day), diabetes, cardiovascular disease history, musculoskeletal disease, chronic lung disease, cancer history, and depression.

After a median follow-up of 13.6 years (range = 0–15), we recorded 4 973 all-cause, 1 581 cardiovascular, and 1 814 cancer deaths. Higher adherence to the SEAD was associated with lower risk of all-cause mortality in the pooled sample [model 2 HR (95% CI) per 1-SD increment = 0.92 (0.89, 0.95)]. All countries showed a similar trend [Spain = 0.93 (0.88, 0.99), Czechia = 0.94 (0.89, 0.99), Poland = 0.89 (0.85, 0.93), United Kingdom = 0.98 (0.89, 1.07); *P* for interaction = 0.16] (*Table 2*, *Figure 1*, Supplementary material online, *Figure S2*).

**Table 2:** Hazard ratios (95% confidence interval) for the association between the Southern European Atlantic Diet and 13.6-year all-cause mortality

	Southern European Atlantic Diet				
	Quartile 1 (lowest)	Quartile 2	Quartile 3	Quartile 4 (highest)	Per 1-SD increment
Pooled sample					
Cases/*n*	1 466/9 981	1 097/8 065	1 079/8 161	1 331/9 710	4 973/35 917
Model 1^a^	Ref.	0.96 (0.89, 1.04)	0.86 (0.80, 0.93)***	0.81 (0.75, 0.88)***	0.91 (0.88, 0.93)***
Model 2^b^	Ref.	0.99 (0.92, 1.07)	0.90 (0.83, 0.98)*	0.85 (0.78, 0.92)***	0.92 (0.89, 0.95)***
Spain					
Cases/*n*	310/3 733	272/3 285	212/2 797	180/2 376	974/12 191
Model 1^a^	Ref.	1.04 (0.88, 1.22)	1.00 (0.84, 1.19)	0.88 (0.73, 1.06)	0.95 (0.89, 1.01)
Model 2^b^	Ref.	1.05 (0.89, 1.23)	0.96 (0.81, 1.15)	0.86 (0.72, 1.04)	0.93 (0.88, 0.99)*
Czechia					
Cases/*n*	521/2 251	323/1 658	374/1 962	478/2 501	1 696/8 372
Model 1^a^	Ref.	0.89 (0.77, 1.02)	0.83 (0.73, 0.95)**	0.85 (0.75, 0.97)*	0.93 (0.89, 0.98)**
Model 2^b^	Ref.	0.89 (0.78, 1.03)	0.86 (0.75, 0.99)*	0.87 (0.77, 1.00)*	0.94 (0.89, 0.99)*
Poland					
Cases/*n*	480/2 121	391/1 888	394/2 279	574/3 656	1 839/9 944
Model 1^a^	Ref.	0.95 (0.83, 1.09)	0.79 (0.69, 0.91)***	0.73 (0.64, 0.82)***	0.86 (0.82, 0.90)***
Model 2^b^	Ref.	1.00 (0.87, 1.14)	0.86 (0.75, 0.98)*	0.79 (0.70, 0.90)***	0.89 (0.85, 0.93)***
UK					
Cases/*n*	155/1 876	111/1 234	99/1 123	99/1 177	464/5 410
Model 1^a^	Ref.	1.08 (0.84, 1.37)	1.02 (0.79, 1.31)	0.91 (0.70, 1.17)	0.94 (0.86, 1.02)
Model 2^b^	Ref.	1.16 (0.91, 1.48)	1.12 (0.87, 1.45)	1.00 (0.77, 1.28)	0.98 (0.89, 1.07)

SD, standard deviation.

^a^Model 1: Cox regression model stratified for country (pooled sample) and adjusted for sex, age, educational level (primary or less, secondary or vocational, university, or no data), and marital status (single, married/cohabiting, divorced/separated or widowed, or no data).

^b^Model 2: As Model 1 and additionally adjusted for tobacco smoking (never, former, current, or no data), leisure time physical activity (hours/week), energy intake (kcal/day), diabetes, cardiovascular disease history, musculoskeletal disease, chronic lung disease, cancer history, and depression.

^*^ *P* < 0.05, **P < 0.01, ***P < 0.001.

**Figure 2:**
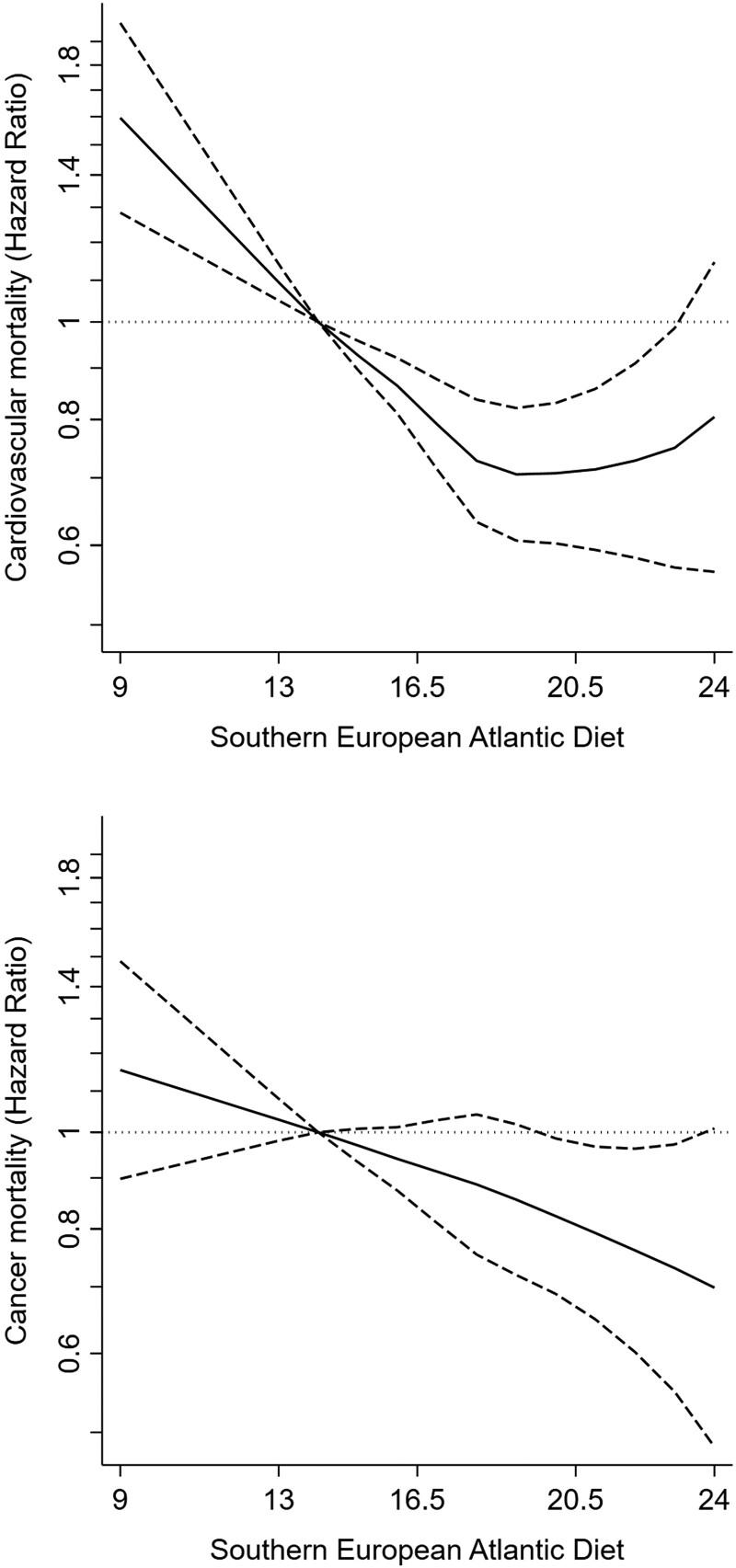
Hazard ratios (95% confidence interval) for the association between the Southern European Atlantic Diet and 13.6-year cardiovascular and cancer mortality in the pooled sample. Cox regression model stratified for country and adjusted as Model 2 in *Table 3*: sex, age, educational level (primary or less, secondary or vocational, university, or no data), marital status (single, married/cohabiting, divorced/separated or widowed, or no data), tobacco smoking (never, former, current, or no data), leisure time physical activity (hours/week), energy intake (kcal/day), diabetes, cardiovascular disease history, musculoskeletal disease, chronic lung disease, cancer history, and depression.

Consistent results were observed for cardiovascular mortality [model 2 HR (95% CI) per 1-SD increment of the SEAD in the pooled sample = 0.91 (0.86, 0.96), Spain = 0.95 (0.83, 1.08), Czechia = 0.92 (0.85, 1.00), Poland = 0.86 (0.79, 0.93), United Kingdom 1.03 = (0.86, 1.23); *P* for country interaction = 0.21] and cancer mortality [pooled sample = 0.94 (0.89, 0.99), Spain = 0.98 (0.87, 1.10), Czechia = 0.92 (0.85, 1.00), Poland = 0.92 (0.85, 0.99), United Kingdom = 1.02 (0.89, 1.16); *P* for country interaction = 0.44] (*Table 3*, *Figure 2*).

**Table 3:** Hazard ratios (95% confidence interval) for the association between the Southern European Atlantic Diet and 13.6-year cardiovascular and cancer mortality

	Southern European Atlantic Diet				
	Quartile 1 (lowest)	Quartile 2	Quartile 3	Quartile 4 (highest)	Per 1-SD increment
**Cardiovascular mortality**					
Pooled sample					
Cases/*n*	500/9 873	322/7 962	329/8 066	430/9 566	1581/35 467
Model 1^a^	Ref.	0.85 (0.74, 0.98)*	0.78 (0.68, 0.90)***	0.78 (0.68, 0.89)***	0.90 (0.85, 0.94)***
Model 2^b^	Ref.	0.88 (0.76, 1.01)	0.81 (0.70, 0.93)**	0.81 (0.70, 0.94)**	0.91 (0.86, 0.96)***
Spain					
Cases/*n*	74/3 699	60/3 249	42/2 771	44/2 345	220/12 064
Model 1^a^	Ref.	0.97 (0.69, 1.36)	0.92 (0.63, 1.34)	0.99 (0.68, 1.44)	0.97 (0.85, 1.10)
Model 2^b^	Ref.	0.99 (0.70, 1.39)	0.85 (0.58, 1.25)	0.99 (0.68, 1.45)	0.95 (0.83, 1.08)
Czechia					
Cases/*n*	213/2 249	115/1 657	148/1 960	173/2 497	649/8 363
Model 1^a^	Ref.	0.79 (0.63, 0.99)*	0.82 (0.66, 1.01)	0.78 (0.64, 0.96)*	0.92 (0.85, 0.99)*
Model 2^b^	Ref.	0.78 (0.62, 0.99)*	0.83 (0.67, 1.03)	0.79 (0.64, 0.98)*	0.92 (0.85, 1.00)*
Poland					
Cases/*n*	174/2 049	119/1 824	119/2 212	185/3 547	597/9 632
Model 1^a^	Ref.	0.82 (0.65, 1.04)	0.68 (0.54, 0.85)**	0.68 (0.55, 0.84)***	0.83 (0.77, 0.90)***
Model 2^b^	Ref.	0.87 (0.69, 1.10)	0.73 (0.58, 0.93)*	0.73 (0.59, 0.91)**	0.86 (0.79, 0.93)***
UK					
Cases/*n*	39/1 876	28/1 232	20/1 123	28/1 177	115/5 408
Model 1^a^	Ref.	1.08 (0.67, 1.76)	0.83 (0.48, 1.41)	1.02 (0.63, 1.66)	0.98 (0.82, 1.17)
Model 2^b^	Ref.	1.19 (0.73, 1.93)	0.93 (0.54, 1.60)	1.13 (0.69, 1.84)	1.03 (0.86, 1.23)
**Cancer mortality**					
Pooled sample					
Cases/*n*	497/9 873	417/7 962	402/8 066	498/9 566	1814/35 467
Model 1^a^	Ref.	1.08 (0.95, 1.23)	0.93 (0.81, 1.06)	0.87 (0.77, 0.99)*	0.92 (0.88, 0.97)***
Model 2^b^	Ref.	1.11 (0.97, 1.27)	0.99 (0.86, 1.13)	0.92 (0.80, 1.06)	0.94 (0.89, 0.99)*
Spain					
Cases/*n*	69/3 699	89/3 249	68/2 771	51/2 345	277/12 064
Model 1^a^	Ref.	1.51 (1.10, 2.07)*	1.34 (0.96, 1.88)	1.01 (0.70, 1.45)	1.00 (0.89, 1.12)
Model 2^b^	Ref.	1.47 (1.07, 2.02)*	1.30 (0.92, 1.82)	0.96 (0.67, 1.39)	0.98 (0.87, 1.10)
Czechia					
Cases/*n*	202/2 249	134/1 657	133/1 960	190/2 497	659/8 363
Model 1^a^	Ref.	0.94 (0.75, 1.16)	0.75 (0.60, 0.94)*	0.86 (0.71, 1.05)	0.91 (0.84, 0.98)*
Model 2^b^	Ref.	0.95 (0.76, 1.18)	0.80 (0.64, 1.00)*	0.90 (0.73, 1.11)	0.92 (0.85, 1.00)*
Poland					
Cases/*n*	159/2 049	134/1 824	144/2 212	209/3 547	646/9 632
Model 1^a^	Ref.	0.97 (0.77, 1.23)	0.86 (0.68, 1.07)	0.78 (0.63, 0.96)*	0.89 (0.82, 0.96)**
Model 2^b^	Ref.	1.02 (0.81, 1.29)	0.94 (0.75, 1.18)	0.87 (0.70, 1.07)	0.92 (0.85, 0.99)*
UK					
Cases/*n*	67/1 876	60/1 232	57/1 123	48/1 177	232/5 408
Model 1 ^a^	Ref.	1.34 (0.95, 1.90)	1.36 (0.95, 1.93)	1.03 (0.71, 1.49)	1.00 (0.88, 1.14)
Model 2 ^b^	Ref.	1.41 (1.00, 2.01)	1.47 (1.03, 2.10)*	1.09 (0.75, 1.58)	1.02 (0.89, 1.16)

SD, standard deviation.

^a^Model 1: Cox regression model stratified for country (pooled sample) and adjusted for sex, age, educational level (primary or less, secondary or vocational, university, or no data), and marital status (single, married/cohabiting, divorced/separated or widowed, or no data).

^b^Model 2: As Model 1 and additionally adjusted for tobacco smoking (never, former, current, or no data), leisure time physical activity (hours/week), energy intake (kcal/day), diabetes, cardiovascular disease history, musculoskeletal disease, chronic lung disease, cancer history, and depression.

^*^ *P* < 0.05, **P < 0.01, ***P < 0.001.

### Other analyses

When analysing the leading causes of cardiovascular and cancer death, the SEAD was associated with reduced mortality from ischaemic heart diseases, malignant neoplasms of digestive organs, and malignant neoplasms of respiratory and intrathoracic organs (see Supplementary material online, *Table S3*).

The protective association between the SEAD and all-cause, cardiovascular, and cancer mortality was of similar magnitude as that found for the AHEI [model 2 HR (95% CI) per 1-SD increment in the pooled sample = 0.95 (0.92, 0.98), 0.95 (0.91, 1.01), and 0.98 (0.93, 1.03), respectively] and the DASH [0.95 (0.92, 0.98), 0.94 (0.89, 0.99), and 0.97 (0.92, 1.02), respectively] (see Supplementary material online, *Tables S4* and *S5*).

Regarding the SEAD food groups, daily consumption of fresh fish (excluding cod), red meat and pork products, dairy, legumes and vegetables, vegetable soup, and whole-grain bread showed a tendency to lower all-cause mortality, contrary to that of potatoes. However, most of these associations were weaker than that of the SEAD as a whole. Consumption of small amounts of wine was associated with lower mortality (see Supplementary material online, *Table S6*).

The results from main analyses held when calculating alternate versions of the SEAD: (i) with reverse scoring for red meat/pork products and potatoes and without scoring wine consumption and (ii) considering total alcohol intake instead of wine consumption. The analyses were also robust to (iii) adjusting for the consumption of common foods not included in the SEAD, (iv) adjusting for hypertension, (v) adjusting for BMI, (vi) not adjusting for morbidity, (vii) omitting the first year of follow-up, (viii) excluding the subjects with severe chronic diseases, and (ix) estimating relative risks of death at 15 years instead of HR (see Supplementary material online, *Table S7*).

We found no evidence that any of the sociodemographic, lifestyle, or morbidity variables included in the models significantly modified the associations between the SEAD and mortality.

## Discussion

In this pooled sample from southern, central, eastern, and western European countries, higher adherence to the SEAD was associated with lower all-cause, cardiovascular, and cancer mortality. Results were not significantly different between countries. The associations were of similar magnitude as those found for the AHEI and the DASH.

### Interpretation

#### Relevant findings from other published studies

The current evidence on the SEAD and health is as follows. In a cross-sectional study in younger adults, higher SEAD adherence was associated with increased concentration of *Bifidobacterium* in faeces—a probiotic genus which could be important for the development of the host immune response.^7^ In two cross-sectional studies, conducted in adolescents and morbidity-free subjects <70 years, respectively, higher SEAD adherence was associated with reduced levels of cardiovascular risk factors, such as C-reactive protein, triglycerides, insulin, insulin resistance, pulse wave velocity, systolic blood pressure, total cholesterol, BMI, and waist circumference.^9–13^ Reductions in the latter three risk factors were reproduced in a controlled trial. Here, 127 families were randomized to a dietary intervention based on the SEAD, which included free provision of seafood, vegetables, dairy, and wine.^14^ In a case-control study, higher SEAD adherence was also linked to lower odds of nonfatal myocardial infarction in adults ≥18 years.^4^

Our results are in line with the other two studies on the SEAD that have previously been conducted in ENRICA. On one hand, higher adherence to the SEAD was cross-sectionally associated with lower levels of C-reactive protein, triglycerides, insulin, insulin resistance, and systolic blood pressure.^8^ On the other hand, 3-year cumulative adherence to the SEAD was associated with a 14% lower risk of 10.9-year all-cause mortality in participants ≥60 years.^15^

#### Possible mechanisms and explanations

Arguably, part of the beneficial associations between the SEAD and health are attributable to its food groups. In the aforementioned studies, those that mostly mediated the beneficial associations between the SEAD and cardiovascular risk factors were fish and legumes/vegetables for C-reactive protein, fish for triglycerides, and cod and legumes/vegetables for blood pressure.^8^ The food groups that contributed most to its inverse association with myocardial infarction were cod, dairy, legumes and vegetables, whole-grain bread, and wine.^4^ Regarding all-cause mortality in older adults, most food groups of the SEAD showed some tendency to risk reduction, but associations were generally weak except for moderate wine consumption.^15^ The same was true in our study as, apart from the latter, only daily consumption of legumes and vegetables was significantly associated with lower cardiovascular mortality (see Supplementary material online, *Table S6*). This is consistent with previous evidence on dietary patterns and suggests that the SEAD may account for the small cumulative effects of individual foods on chronic disease and complex interactions between food groups.^33^

Nevertheless, not all the SEAD food groups might be optimal from the overall health perspective. Two recent dose–response meta-analyses showed a beneficial association of fish, legumes, vegetables, and whole-grain consumption with all-cause mortality, no association for dairy and potatoes, and a detrimental one for red and processed meat consumption.^18,19^ Moreover, red meat and pork products have been negatively associated with cardiometabolic disease and cancer,^16,17^ while potatoes may increase the risk of type 2 diabetes.^19^ This evidence is in line with the sensitivity analyses conducted by us and others, as daily potato consumption was associated with higher all-cause mortality (see Supplementary material online, *Table S6*), and a SEAD calculated with reverse scoring for red meat/pork products and potatoes was more strongly associated with lower odds of myocardial infarction than the traditional SEAD.^4^

Contrary to our observations, there is increasing evidence of no mortality benefits conferred by moderate alcohol intake.^20,21^ On one hand, the association of wine and mortality might be different to that of beer or spirits, partially because it is drunk more frequently (when consuming the same amount) than the said beverages and rarely entails binge drinking.^20^ On the other hand, the SEAD scoring for wine consumption does not account for the abstainer bias, the healthy drinker/survivor bias, or reverse causation,^21^ which may explain the beneficial association of this food group with mortality in our study—note that 59% of the subjects who were given 0 points for wine consumption were never or former drinkers.

Together with the potential concerns raised by red meat/pork products and potatoes, those of alcohol consumption leave the door open for optimizing the SEAD to be used in public health interventions, either in its countries of origin or elsewhere. It is of note that a SEAD calculated with reverse scoring for red meat/pork products and potatoes and without scoring wine was also associated with lower all-cause mortality (see Supplementary material online, *Table S7*).

### Generalizability

Though there were no significant differences in study associations across countries, the relationship between the SEAD and all-cause and cause-specific mortality was consistently weaker in the United Kingdom (*Tables 2* and *3*). All-cause mortality looked the epitome of these differences, as the SEAD was associated with lower mortality in Spain, Czechia, and Poland, but not in the United Kingdom. Any explanation for these findings must be conjectural.

On one hand, the consumption of the SEAD food groups differed by country. Note that equal SEAD scores could be obtained from substantially different combinations of food consumption, which in turn may have opposed associations with mortality. For instance, potato consumption was highest in the United Kingdom, and this food group was the only one showing a tendency to increased all-cause and cancer mortality (see Supplementary material online, *Table S2*).

On the other hand, these differences in study associations may reflect reverse causation, as 30% of our subjects suffered from at least one severe chronic disease at baseline (diabetes, cardiovascular disease, chronic lung disease, or cancer). Since these subjects were under increased risk of death and their adherence to the SEAD differed from that of their disease-free counterparts at the pooled sample and country levels (*Table 1*, Supplementary material online, *Table S1*), this could have biased the study results if illness had led individuals to change their dietary habits. Specifically, diagnoses of several somatic diseases may be related to improvements in diet quality,^34,35^ while current depression is associated with poorer dietary habits.^36^ In line with this evidence, we observed that individuals in the highest quartiles of the SEAD had a higher prevalence of diabetes, cardiovascular disease, musculoskeletal disease, and chronic lung disease, while they suffered less from depression (*Table 1*).

It is therefore reassuring that excluding the subjects with severe chronic diseases from the analyses strengthened most associations between the SEAD and mortality and virtually made between-country variation disappear. This convergence towards the pooled estimates was especially marked for all-cause mortality (*P* for country interaction = 0.77), as even the one-time outliers (i.e. United Kingdom) reached significance (see Supplementary material online, *Table S7*).

The fact that our study included participants aged 18–96 years, almost equal numbers of men and women, from diverse educational backgrounds, a wide array of lifestyles, and with and without chronic diseases, likely strengthens the generalizability of our findings. In addition, we observed no significant interactions for any of the sociodemographic, lifestyle, and morbidity variables included in the analyses. It should be noted, however, that response rates in the population-based cohorts were not always optimal,^24,25^ and participants in the Whitehall II study were systematically different to the general population.^29^ Moreover, most of the subjects from Spain and the United Kingdom were white/Caucasians (95.9%) and we lacked data on ethnicity for Czechia and Poland. This warrants caution when extrapolating our results to the targeted European countries and multiethnic/multiracial populations.

### Limitations

A further limitation is the scoring method used to estimate the adherence to the SEAD. On one hand, there was some evidence of ceiling effect for red meat/pork product, dairy, and legume/vegetable consumption, suggesting that only a subset of food groups contributed to the protective association of the SEAD with mortality. On the other hand, most food groups were scored according to the frequency of consumption (i.e. more points were given to those consuming more servings), and therefore, the SEAD showed a positive correlation with energy intake (*Table 1*).

Some limitations in dietary assessment should also be acknowledged. In previous validation studies, the correlation of ENRICA’s diet history and Whitehall II’s FFQ with seven 24 h recalls was moderate (e.g. 0.76 and 0.34 for energy, 0.49 and 0.50 for fibre, and 0.69 and 0.81 for alcohol).^26,30^ Despite no such data being available for HAPIEE’s FFQ, it was based on Whitehall II’s, so similar results would have been expected.^37^ Furthermore, the correlation of the diet history and FFQs with plasma biomarkers of food consumption was low (e.g. 0.46 and 0.30 for eicosapentaenoic acid in ENRICA and Whitehall II, 0.28 and 0.32 for vitamin C in ENRICA and HAPIEE, and 0.17 and 0.20 for β-carotene in HAPIEE and Whitehall II), though similar to those of other self-reported methods used to measure habitual diet.^26,30,37^ A potential strategy to overcome this limitation would have been to use repeated measurements of diet in the analyses, but we lacked such data—note that, in ENRICA, subjects <60 years were never followed, while in HAPIEE, no dietary data were collected in the follow-up waves. In any case, the inability to measure the true value of a dietary exposure would habitually bias the study results towards the null, meaning that we would likely be underestimating the true association between the SEAD and mortality.^38^

Finally, there is potential for non-response bias (because the proportion of non-responders was higher in the lowest quartile of the SEAD) and residual confounding, as many covariates were likely to be measured with some error, and some potential confounders could not be accounted for. First, cardiovascular disease history, musculoskeletal disease, chronic lung disease, and cancer history were self-reported in ENRICA and HAPIEE. Second, we lacked data on leisure time physical activity intensity and sedentary behaviours in HAPIEE, and both are associated with mortality independently of total physical activity time.^39,40^ Third, though economic disparities may influence both diet and health,^41^ we could not adjust the analyses for material deprivation because this information was not collected in ENRICA. When taking these limitations together, the real range of uncertainty in HRs could be larger than that reflected in CIs, for when exposures and confounders are measured with various degrees of precision, the adjusted estimates could be biased in any direction.^42^ It is encouraging, though, to see that modelling the SEAD as a continuous (per 1-SD increment) and a categorical (quartiles) variable rendered similar results, as did minimally adjusted and fully adjusted models (even those accounting for habitual foods not included in the SEAD). Moreover, the study associations were not significantly different between countries, even if the distribution of potential confounders (both measured and unmeasured) between SEAD categories was likely unequal (see Supplementary material online, *Table S1*).

## Conclusions

In a pooled sample from southern, central, eastern, and western European countries, higher adherence to the SEAD was associated with lower 13.6-year mortality from any cause, cardiovascular disease, and cancer. These associations were of moderate magnitude but consistent in main and sensitivity analyses. Given that diet is often measured with some error, can change over time, and its effects on health could be cumulative, evidence from studies with repeated measurements of diet would be desirable.

These findings may support the development of dietary guidelines for northwestern Spain and northern Portugal based on their traditional diet. Dietary recommendations for central, eastern, and western European countries could set their sights on the SEAD food groups that are culturally rooted in these regions (e.g. vegetable soups, dairy, and brown bread). The fact that the study associations were similar as those found for existing healthy dietary patterns, such as the AHEI and the DASH, suggests that rather different diets could confer comparable benefits on health.

## Supplementary material

Supplementary material is available at *European Journal of Preventive Cardiology*.

## Supplementary Material

zwad370_Supplementary_Data

## Data Availability

The ENRICA, HAPIEE, and Whitehall II data sets used and/or analysed during the current study are available from the study authors on reasonable request.
