# Supplementary material for: The Southern European Atlantic diet and all-cause and cause-specific mortality: a European multicohort study
Source: Eur J Prev Cardiol. 2023 Dec 15;31(3):358–67. doi: 10.1093/eurjpc/zwad370 (PMC10873144; doi:10.1093/eurjpc/zwad370)
Supplement: zwad370_Supplementary_Data [file zwad370_supplementary_data.docx]

**Appendix S1.** Categorization of the Southern European Atlantic Diet.

Quartiles: Spain (Quartile 1, 8 to 14; Quartile 2, 15; Quartile 3, 16; Quartile 4, 17 to 21), Czechia (Quartile 1, 8 to 16; Quartile 2, 17; Quartile 3, 18; Quartile 4, 19 to 24), Poland (Quartile 1, 9 to 16; Quartile 2, 17; Quartile 3, 18; Quartile 4, 19 to 23), UK (Quartile 1, 8 to 17; Quartile 2, 18; Quartile 3, 19; Quartile 3, 20 to 23).

1-Standard Deviation increment: Spain, 1.53; Czechia, 1.80; Poland, 1.74; UK, 1.78.

Restricted cubic spline knots: Spain (knot 1, 13; knot 2, 15; knot 3, 17), Czechia (knot 1, 15; knot 2, 18; knot 3, 20), Poland (knot 1, 16; knot 2, 18; knot 3, 20), UK (knot 1, 16; knot 2, 18; knot 3, 20), reference (14).

**Table S1.** Characteristics of the study individuals, by country.

|  | **Spain** | **Czechia** | **Poland** | **UK** |
| --- | --- | --- | --- | --- |
| n | 12191 | 8372 | 9944 | 5410 |
| Sex-Male (%) | 5772 (47.3) | 3912 (46.7) | 4834 (48.6) | 3855 (71.3)^*^ |
| Age (years) | 47.9 (16.7) | 58.3 (7.14) | 57.6 (6.97) | 56.0 (6.08)^*^ |
| Educational level (%) |  |  |  |  |
| Primary or less | 3528 (28.9) | 1029 (12.3) | 1121 (11.3) | 0 (0)^*^ |
| Secondary or vocational | 5110 (41.9) | 6156 (73.5) | 5971 (60.0) | 3407 (63.0) |
| University | 3516 (28.8) | 1148 (13.7) | 2844 (28.6) | 1917 (35.4) |
| No data | 37 (0.30) | 39 (0.47) | 8 (0.080) | 86 (1.59) |
| Marital status (%) |  |  |  |  |
| Single | 3058 (25.1) | 218 (2.60) | 534 (5.37) | 614 (11.3)^*^ |
| Married/cohabiting | 7770 (63.7) | 6329 (75.6) | 7586 (76.3) | 4065 (75.1) |
| Divorced/separated or widowed | 1180 (9.68) | 1792 (21.4) | 1801 (18.1) | 500 (9.24) |
| No data | 183 (1.50) | 33 (0.39) | 23 (0.23) | 231 (4.27) |
| Tobacco smoking (%) |  |  |  |  |
| Never | 5791 (47.5) | 3634 (43.4) | 3906 (39.3) | 2678 (49.5)^*^ |
| Former | 3083 (25.3) | 2454 (29.3) | 2812 (28.3) | 2206 (40.8) |
| Current | 3275 (26.9) | 2179 (26.0) | 3198 (32.2) | 482 (8.91) |
| No data | 42 (0.34) | 105 (1.25) | 28 (0.28) | 44 (0.81) |
| Physical activity (hours/week) | 21.9 (14.9) | 17.3 (14.4) | 18.2 (13.0) | 21.4 (15.9)^*^ |
| Energy intake (kcal/day) | 2195 (642) | 2024 (818) | 2151 (670) | 2211 (665)^*^ |
| Diabetes (%) | 805 (6.60) | 1424 (17.0) | 1374 (13.8) | 193 (3.57)^*^ |
| Cardiovascular disease history (%) | 270 (2.21) | 1100 (13.1) | 2299 (23.1) | 781 (14.4)^*^ |
| Musculoskeletal disease (%) | 2619 (21.5) | 4594 (54.9) | 6911 (69.5) | 1860 (34.4)^*^ |
| Chronic lung disease (%) | 745 (6.11) | 1286 (15.4) | 1378 (13.9) | 636 (11.8)^*^ |
| Cancer history (%) | 131 (1.07) | 509 (6.08) | 470 (4.73) | 235 (4.34)^*^ |
| Depression (%) | 936 (7.68) | 915 (10.9) | 1623 (16.3) | 752 (13.9)^*^ |

Values are numbers (%) or means (standard deviations).

^*^P-value <0.05 for differences in means (ANOVA) or proportions (Pearson’s chi-squared) across countries.

**Table S2.** Consumption of the Southern European Atlantic Diet food groups.

|  | **Spain** | **Czechia** | **Poland** | **UK** | **Total** |
| --- | --- | --- | --- | --- | --- |
| n | 12191 | 8372 | 9944 | 5410 | 35917 |
| **Fresh fish (excluding cod)** |  |  |  |  |  |
| Less than weekly | 1373 (11.3) | 5907 (70.6) | 3090 (31.1) | 2804 (51.8)^*^ | 13174 (36.7) |
| 1 to 6 times/week | 9471 (77.7) | 2448 (29.2) | 6742 (67.8) | 2574 (47.6) | 21235 (59.1) |
| Daily | 1347 (11.0) | 17 (0.20) | 112 (1.13) | 32 (0.59) | 1508 (4.20) |
| **Cod** |  |  |  |  |  |
| Less than weekly | 11621 (95.3) | 7011 (83.7) | 5649 (56.8) | 1749 (32.3)^*^ | 26030 (72.5) |
| 1 to 6 times/week | 568 (4.66) | 1351 (16.1) | 4287 (43.1) | 3650 (67.5) | 9856 (27.4) |
| Daily | 2 (0.02) | 10 (0.12) | 8 (0.08) | 11 (0.20) | 31 (0.09) |
| **Red meat and pork products** |  |  |  |  |  |
| Less than weekly | 913 (7.49) | 123 (1.47) | 96 (0.97) | 521 (9.63)^*^ | 1653 (4.60) |
| 1 to 6 times/week | 6702 (55.0) | 4421 (52.8) | 4515 (45.4) | 3582 (66.2) | 19220 (53.5) |
| Daily | 4576 (37.5) | 3828 (45.7) | 5333 (53.6) | 1307 (24.2) | 15044 (41.9) |
| **Dairy** |  |  |  |  |  |
| Less than weekly | 246 (2.02) | 75 (0.90) | 135 (1.36) | 42 (0.78)^*^ | 498 (1.39) |
| 1 to 6 times/week | 2424 (19.9) | 1759 (21.0) | 2173 (21.9) | 419 (7.74) | 6775 (18.9) |
| Daily | 9521 (78.1) | 6538 (78.1) | 7636 (76.8) | 4949 (91.5) | 28644 (79.8) |
| **Legumes and vegetables** |  |  |  |  |  |
| Less than weekly | 102 (0.84) | 37 (0.44) | 12 (0.12) | 21 (0.39)^*^ | 172 (0.48) |
| 1 to 6 times/week | 2833 (23.2) | 371 (4.43) | 302 (3.04) | 73 (1.35) | 3579 (9.96) |
| Daily | 9256 (75.9) | 7964 (95.1) | 9630 (96.8) | 5316 (98.3) | 32166 (89.6) |
| **Vegetable soup** |  |  |  |  |  |
| Less than weekly | 11148 (91.4) | 1705 (20.4) | 564 (5.67) | 3558 (65.8)^*^ | 16975 (47.3) |
| 1 to 6 times/week | 1009 (8.28) | 5780 (69.0) | 8730 (87.8) | 1788 (33.0) | 17307 (48.2) |
| Daily | 34 (0.28) | 887 (10.6) | 650 (6.54) | 64 (1.18) | 1635 (4.55) |
| **Potatoes** |  |  |  |  |  |
| Less than weekly | 3519 (28.9) | 103 (1.23) | 336 (3.38) | 126 (2.33)^*^ | 4084 (11.4) |
| 1 to 6 times/week | 8512 (69.8) | 7006 (83.7) | 7852 (79.0) | 2964 (54.8) | 26334 (73.3) |
| Daily | 160 (1.31) | 1263 (15.1) | 1756 (17.7) | 2320 (42.9) | 5499 (15.3) |
| **Whole-grain bread** |  |  |  |  |  |
| Less than weekly | 10264 (84.2) | 1549 (18.5) | 3510 (35.3) | 695 (12.8)^*^ | 16018 (44.6) |
| 1 to 6 times/week | 801 (6.57) | 1901 (22.7) | 1715 (17.2) | 1665 (30.8) | 6082 (16.9) |
| Daily | 1126 (9.24) | 4922 (58.8) | 4719 (47.5) | 3050 (56.4) | 13817 (38.5) |
| **Wine** |  |  |  |  |  |
| 0 or >1 glass/day (women),  0 or >2 glasses/day (men) | 8608 (70.6) | 4275 (51.1) | 7966 (80.1) | 1664 (30.8)^*^ | 22513 (62.7) |
| ≥0 to 1 glass/day (women),  ≥0 to 2 glasses/day (men) | 3583 (29.4) | 4097 (48.9) | 1978 (19.9) | 3746 (69.2) | 13404 (37.3) |

Values are numbers (%).

^*^P-value <0.05 for differences in proportions (Pearson’s chi-squared) across countries.

**Table S3.** Hazard Ratios (95% confidence interval) for the association between the Southern European Atlantic Diet and 13.6-year leading causes of cardiovascular and cancer mortality in the pooled sample.

|  | **Southern European Atlantic Diet** | | | | |
| --- | --- | --- | --- | --- | --- |
|  | **Quartile 1 (lowest)** | **Quartile 2** | **Quartile 3** | **Quartile 4 (highest)** | **Per 1-SD increment** |
| Cardiovascular mortality |  |  |  |  |  |
| **Ischemic heart diseases** |  |  |  |  |  |
| Cases/n | 261/9785 | 162/7899 | 189/7991 | 233/9461 | 845/35136 |
| Model 1 ^a^ | Ref. | 0.85 (0.70,1.03) | 0.85 (0.70,1.03) | 0.80 (0.67,0.96)* | 0.92 (0.87,0.99)* |
| Model 2 ^b^ | Ref. | 0.86 (0.70,1.04) | 0.87 (0.72,1.06) | 0.79 (0.65,0.96)* | 0.92 (0.86,0.99)* |
| **Cerebrovascular diseases** |  |  |  |  |  |
| Cases/n | 83/9785 | 75/7899 | 70/7991 | 70/9461 | 298/35136 |
| Model 1 ^a^ | Ref. | 1.19 (0.87,1.63) | 1.00 (0.72,1.37) | 0.78 (0.57,1.08) | 0.89 (0.80,1.00)* |
| Model 2 ^b^ | Ref. | 1.25 (0.91,1.72) | 1.05 (0.76,1.47) | 0.84 (0.59,1.20) | 0.92 (0.81,1.04) |
| **Heart failure** |  |  |  |  |  |
| Cases/n | 35/9785 | 20/7899 | 20/7991 | 29/9461 | 104/35136 |
| Model 1 ^a^ | Ref. | 0.81 (0.47,1.40) | 0.79 (0.46,1.38) | 0.99 (0.60,1.64) | 1.00 (0.83,1.21) |
| Model 2 ^b^ | Ref. | 0.86 (0.49,1.51) | 0.89 (0.50,1.59) | 1.17 (0.66,2.06) | 1.07 (0.86,1.33) |
| Cancer mortality |  |  |  |  |  |
| **Digestive organs** |  |  |  |  |  |
| Cases/n | 174/9785 | 159/7899 | 139/7991 | 170/9461 | 642/35136 |
| Model 1 ^a^ | Ref. | 1.20 (0.96,1.48) | 0.93 (0.75,1.17) | 0.88 (0.71,1.09) | 0.92 (0.85,0.99)* |
| Model 2 ^b^ | Ref. | 1.20 (0.97,1.49) | 0.95 (0.76,1.20) | 0.89 (0.70,1.12) | 0.91 (0.84,1.00)* |
| **Respiratory and intrathoracic organs** |  |  |  |  |  |
| Cases/n | 146/9785 | 84/7899 | 95/7991 | 113/9461 | 438/35136 |
| Model 1 ^a^ | Ref. | 0.74 (0.57,0.97)* | 0.72 (0.56,0.94)* | 0.64 (0.50,0.82)*** | 0.83 (0.76,0.91)*** |
| Model 2 ^b^ | Ref. | 0.80 (0.61,1.05) | 0.83 (0.64,1.09) | 0.74 (0.56,0.97)* | 0.88 (0.79,0.97)* |
| **Lymphoid, hematopoietic and related tissue** |  |  |  |  |  |
| Cases/n | 87/9785 | 64/7899 | 55/7991 | 70/9461 | 276/35136 |
| Model 1 ^a^ | Ref. | 0.97 (0.70,1.34) | 0.76 (0.54,1.07) | 0.75 (0.54,1.03) | 0.87 (0.77,0.97)* |
| Model 2 ^b^ | Ref. | 1.06 (0.76,1.47) | 0.86 (0.60,1.22) | 0.88 (0.62,1.25) | 0.92 (0.81,1.05) |

Cardiovascular mortality: ischemic heart diseases -ICD-10 codes ranging from I20 to I25 and ICD-9 from 410 to 414-, cerebrovascular diseases -I60 to I69 and 430 to 438-, and heart failure -I50 and 428.

Cancer mortality: digestive organs -ICD-10 codes ranging from C15 to C26 and ICD-9 from 150 to 159-, respiratory and intrathoracic organs -C30 to C39 and 160 to 165-, and lymphoid, hematopoietic and related tissue -C81 to C96 and 200 to 208.

*p<0.05. **p<0.01. ***p<0.001. SD = Standard Deviation.

^a^ Model 1: Cox regression model stratified for country, and adjusted for sex, age, educational level (primary or less, secondary or vocational, university, or no data), and marital status (single, married/cohabiting, divorced/separated or widowed, or no data).

^b^ Model 2: As Model 1 and additionally adjusted for tobacco smoking (never, former, current, or no data), leisure-time physical activity (hours/week), energy intake (kcal/day), diabetes, cardiovascular disease history, musculoskeletal disease, chronic lung disease, cancer history and depression.

**Table S4.** Hazard Ratios (95% confidence interval) for the association between the Alternate Healthy Eating Index and 13.6-year mortality.

|  | **Alternate Healthy Eating Index ^a^** | | | | |
| --- | --- | --- | --- | --- | --- |
|  | **Quartile 1 (lowest)** | **Quartile 2** | **Quartile 3** | **Quartile 4 (highest)** | **Per 1-SD increment** |
| All-cause mortality |  |  |  |  |  |
| **Pooled sample** |  |  |  |  |  |
| Cases/n | 1149/8980 | 1280/8979 | 1248/8980 | 1296/8978 | 4973/35917 |
| Model 1 ^b^ | Ref. | 1.02 (0.94,1.11) | 0.97 (0.89,1.05) | 0.89 (0.81,0.97)** | 0.95 (0.92,0.98)*** |
| Model 2 ^c^ | Ref. | 1.01 (0.93,1.10) | 0.96 (0.88,1.05) | 0.89 (0.82,0.97)* | 0.95 (0.92,0.98)** |
| **Spain** |  |  |  |  |  |
| Cases/n | 148/3048 | 231/3048 | 265/3048 | 330/3047 | 974/12191 |
| Model 1 ^b^ | Ref. | 1.01 (0.81,1.26) | 0.95 (0.77,1.18) | 0.75 (0.60,0.92)** | 0.88 (0.82,0.95)*** |
| Model 2 ^c^ | Ref. | 1.01 (0.81,1.26) | 1.00 (0.81,1.24) | 0.82 (0.67,1.01) | 0.92 (0.86,0.99)* |
| **Czechia** |  |  |  |  |  |
| Cases/n | 436/2093 | 443/2093 | 421/2093 | 396/2093 | 1696/8372 |
| Model 1 ^b^ | Ref. | 0.99 (0.86,1.13) | 0.98 (0.85,1.12) | 0.90 (0.78,1.03) | 0.95 (0.91,1.00) |
| Model 2 ^c^ | Ref. | 0.99 (0.87,1.13) | 0.98 (0.86,1.12) | 0.92 (0.80,1.05) | 0.96 (0.92,1.01) |
| **Poland** |  |  |  |  |  |
| Cases/n | 439/2486 | 488/2486 | 452/2486 | 460/2486 | 1839/9944 |
| Model 1 ^b^ | Ref. | 1.11 (0.96,1.28) | 1.00 (0.87,1.16) | 1.01 (0.87,1.17) | 1.00 (0.95,1.05) |
| Model 2 ^c^ | Ref. | 1.06 (0.92,1.22) | 0.95 (0.82,1.10) | 0.94 (0.81,1.09) | 0.97 (0.92,1.03) |
| **UK** |  |  |  |  |  |
| Cases/n | 126/1353 | 118/1352 | 110/1353 | 110/1352 | 464/5410 |
| Model 1 ^b^ | Ref. | 0.91 (0.71,1.17) | 0.81 (0.63,1.04) | 0.83 (0.64,1.07) | 0.90 (0.82,0.99)* |
| Model 2 ^c^ | Ref. | 0.95 (0.74,1.23) | 0.86 (0.67,1.12) | 0.90 (0.70,1.16) | 0.93 (0.85,1.02) |

**Table S4** (continued)

|  | **Alternate Healthy Eating Index ^a^** | | | | |
| --- | --- | --- | --- | --- | --- |
|  | **Quartile 1 (lowest)** | **Quartile 2** | **Quartile 3** | **Quartile 4 (highest)** | **Per 1-SD increment** |
| Cardiovascular mortality |  |  |  |  |  |
| **Pooled sample** |  |  |  |  |  |
| Cases/n | 359/8876 | 400/8862 | 417/8880 | 405/8849 | 1581/35467 |
| Model 1 ^b^ | Ref. | 1.04 (0.90,1.20) | 1.05 (0.91,1.21) | 0.93 (0.81,1.08) | 0.96 (0.91,1.01) |
| Model 2 ^c^ | Ref. | 1.01 (0.87,1.16) | 1.02 (0.89,1.18) | 0.91 (0.79,1.06) | 0.95 (0.91,1.01) |
| **Spain** |  |  |  |  |  |
| Cases/n | 30/3029 | 51/3014 | 57/3019 | 82/3002 | 220/12064 |
| Model 1 ^b^ | Ref. | 1.09 (0.69,1.71) | 0.96 (0.62,1.49) | 0.85 (0.55,1.29) | 0.87 (0.76,1.00) |
| Model 2 ^c^ | Ref. | 1.06 (0.67,1.66) | 0.99 (0.63,1.54) | 0.92 (0.60,1.40) | 0.91 (0.79,1.05) |
| **Czechia** |  |  |  |  |  |
| Cases/n | 172/2088 | 164/2091 | 165/2092 | 148/2092 | 649/8363 |
| Model 1 ^b^ | Ref. | 0.93 (0.75,1.15) | 0.97 (0.78,1.20) | 0.85 (0.68,1.05) | 0.93 (0.86,1.00) |
| Model 2 ^c^ | Ref. | 0.92 (0.74,1.15) | 0.96 (0.77,1.19) | 0.85 (0.68,1.06) | 0.93 (0.86,1.01) |
| **Poland** |  |  |  |  |  |
| Cases/n | 130/2407 | 158/2406 | 165/2416 | 144/2403 | 597/9632 |
| Model 1 ^b^ | Ref. | 1.17 (0.93,1.48) | 1.19 (0.95,1.50) | 1.06 (0.84,1.35) | 1.04 (0.95,1.12) |
| Model 2 ^c^ | Ref. | 1.10 (0.87,1.39) | 1.10 (0.87,1.39) | 0.95 (0.74,1.21) | 1.00 (0.92,1.08) |
| **UK** |  |  |  |  |  |
| Cases/n | 27/1352 | 27/1351 | 30/1353 | 31/1352 | 115/5408 |
| Model 1 ^b^ | Ref. | 0.98 (0.57,1.66) | 1.02 (0.60,1.71) | 1.08 (0.65,1.81) | 0.95 (0.79,1.14) |
| Model 2 ^c^ | Ref. | 1.03 (0.60,1.75) | 1.07 (0.64,1.80) | 1.16 (0.69,1.95) | 0.98 (0.82,1.18) |

**Table S4** (continued)

|  | **Alternate Healthy Eating Index ^a^** | | | | |
| --- | --- | --- | --- | --- | --- |
|  | **Quartile 1 (lowest)** | **Quartile 2** | **Quartile 3** | **Quartile 4 (highest)** | **Per 1-SD increment** |
| Cancer mortality |  |  |  |  |  |
| **Pooled sample** |  |  |  |  |  |
| Cases/n | 427/8876 | 482/8862 | 435/8880 | 470/8849 | 1814/35467 |
| Model 1 ^b^ | Ref. | 1.05 (0.92,1.19) | 0.90 (0.79,1.03) | 0.92 (0.80,1.05) | 0.97 (0.92,1.01) |
| Model 2 ^c^ | Ref. | 1.06 (0.93,1.21) | 0.92 (0.81,1.06) | 0.95 (0.83,1.09) | 0.98 (0.93,1.03) |
| **Spain** |  |  |  |  |  |
| Cases/n | 43/3029 | 69/3014 | 78/3019 | 87/3002 | 277/12064 |
| Model 1 ^b^ | Ref. | 1.06 (0.72,1.55) | 0.92 (0.63,1.34) | 0.75 (0.52,1.08) | 0.92 (0.81,1.04) |
| Model 2 ^c^ | Ref. | 1.10 (0.75,1.61) | 1.00 (0.69,1.45) | 0.84 (0.58,1.22) | 0.96 (0.85,1.09) |
| **Czechia** |  |  |  |  |  |
| Cases/n | 161/2088 | 171/2091 | 156/2092 | 171/2092 | 659/8363 |
| Model 1 ^b^ | Ref. | 1.02 (0.82,1.27) | 0.96 (0.77,1.19) | 1.03 (0.83,1.28) | 1.01 (0.93,1.09) |
| Model 2 ^c^ | Ref. | 1.04 (0.84,1.29) | 0.98 (0.78,1.22) | 1.08 (0.87,1.34) | 1.03 (0.95,1.11) |
| **Poland** |  |  |  |  |  |
| Cases/n | 163/2407 | 178/2406 | 147/2416 | 158/2403 | 646/9632 |
| Model 1 ^b^ | Ref. | 1.07 (0.87,1.33) | 0.85 (0.68,1.07) | 0.93 (0.75,1.16) | 0.96 (0.89,1.04) |
| Model 2 ^c^ | Ref. | 1.07 (0.86,1.32) | 0.84 (0.67,1.05) | 0.92 (0.73,1.15) | 0.96 (0.88,1.04) |
| **UK** |  |  |  |  |  |
| Cases/n | 60/1352 | 64/1351 | 54/1353 | 54/1352 | 232/5408 |
| Model 1 ^b^ | Ref. | 1.03 (0.72,1.46) | 0.82 (0.57,1.19) | 0.85 (0.59,1.22) | 0.92 (0.81,1.05) |
| Model 2 ^c^ | Ref. | 1.07 (0.75,1.52) | 0.89 (0.61,1.28) | 0.91 (0.63,1.32) | 0.95 (0.83,1.08) |

*p<0.05. **p<0.01. ***p<0.001. SD=Standard Deviation.

^a^ Quartiles of the Alternate Healthy Eating Index: Spain (Quartile 1, 14.0 to 50.4; Quartile 2, 50.4 to 57.8; Quartile 3, 57.8 to 65.1; Quartile 4, 65.1 to 93.8), Czechia (Quartile 1, 13.2 to 34.3; Quartile 2, 34.3 to 40.5; Quartile 3, 40.5 to 46.6; Quartile 4, 46.6 to 71.7), Poland (Quartile 1, 13.2 to 32.1; Quartile 2, 32.2 to 37.2; Quartile 3, 37.2 to 42.6; Quartile 4, 42.6 to 72.7), UK (Quartile 1, 20.6 to 43.3; Quartile 2, 43.3 to 49.4; Quartile 3, 49.4 to 55.3; Quartile 4, 55.3 to 81.0).

1-SD increment: Spain, 10.9; Czechia, 8.90; Poland, 7.81; UK, 8.77.

^b^ Model 1: Cox regression model stratified for country (pooled sample), and adjusted for sex, age, educational level (primary or less, secondary or vocational, university, or no data), and marital status (single, married/cohabiting, divorced/separated or widowed, or no data).

^c^ Model 2: As Model 1 and additionally adjusted for tobacco smoking (never, former, current, or no data), leisure-time physical activity (hours/week), energy intake (kcal/day), diabetes, cardiovascular disease history, musculoskeletal disease, chronic lung disease, cancer history and depression.

**Table S5.** Hazard Ratios (95% confidence interval) for the association between the Dietary Approaches to Stop Hypertension and 13.6-year mortality.

|  | **Dietary Approaches to Stop Hypertension ^a^** | | | | |
| --- | --- | --- | --- | --- | --- |
|  | **Quartile 1 (lowest)** | **Quartile 2** | **Quartile 3** | **Quartile 4 (highest)** | **Per 1-SD increment** |
| All-cause mortality |  |  |  |  |  |
| **Pooled sample** |  |  |  |  |  |
| Cases/n | 1399/10388 | 1268/9444 | 1263/8461 | 1043/7624 | 4973/35917 |
| Model 1 ^b^ | Ref. | 0.90 (0.83,0.98)* | 0.92 (0.84,0.99)* | 0.78 (0.71,0.85)*** | 0.92 (0.89,0.95)*** |
| Model 2 ^c^ | Ref. | 0.93 (0.85,1.00) | 0.96 (0.88,1.04) | 0.84 (0.77,0.92)*** | 0.95 (0.92,0.98)*** |
| **Spain** |  |  |  |  |  |
| Cases/n | 141/3268 | 252/3242 | 299/2951 | 282/2730 | 974/12191 |
| Model 1 ^b^ | Ref. | 0.93 (0.75,1.17) | 0.89 (0.71,1.10) | 0.78 (0.63,0.98)* | 0.93 (0.86,1.00)* |
| Model 2 ^c^ | Ref. | 0.92 (0.73,1.15) | 0.93 (0.74,1.15) | 0.85 (0.68,1.06) | 0.96 (0.89,1.04) |
| **Czechia** |  |  |  |  |  |
| Cases/n | 538/2612 | 415/2071 | 398/1911 | 345/1778 | 1696/8372 |
| Model 1 ^b^ | Ref. | 0.93 (0.82,1.06) | 0.95 (0.83,1.08) | 0.85 (0.74,0.97)* | 0.94 (0.89,0.99)* |
| Model 2 ^c^ | Ref. | 0.98 (0.86,1.11) | 0.98 (0.86,1.11) | 0.91 (0.79,1.04) | 0.96 (0.92,1.01) |
| **Poland** |  |  |  |  |  |
| Cases/n | 570/2970 | 486/2702 | 465/2324 | 318/1948 | 1839/9944 |
| Model 1 ^b^ | Ref. | 0.89 (0.78,1.01) | 0.94 (0.82,1.08) | 0.70 (0.60,0.82)*** | 0.90 (0.85,0.95)*** |
| Model 2 ^c^ | Ref. | 0.90 (0.79,1.03) | 0.99 (0.86,1.13) | 0.75 (0.64,0.87)*** | 0.92 (0.87,0.97)** |
| **UK** |  |  |  |  |  |
| Cases/n | 150/1538 | 115/1429 | 101/1275 | 98/1168 | 464/5410 |
| Model 1 ^b^ | Ref. | 0.78 (0.61,0.99)* | 0.78 (0.61,1.01) | 0.77 (0.60,1.00) | 0.90 (0.82,0.99)* |
| Model 2 ^c^ | Ref. | 0.81 (0.63,1.03) | 0.86 (0.67,1.11) | 0.87 (0.68,1.13) | 0.95 (0.87,1.05) |

**Table S5** (continued)

|  | **Dietary Approaches to Stop Hypertension ^a^** | | | | |
| --- | --- | --- | --- | --- | --- |
|  | **Quartile 1 (lowest)** | **Quartile 2** | **Quartile 3** | **Quartile 4 (highest)** | **Per 1-SD increment** |
| Cardiovascular mortality |  |  |  |  |  |
| **Pooled sample** |  |  |  |  |  |
| Cases/n | 464/10257 | 401/9354 | 390/8340 | 326/7516 | 1581/35467 |
| Model 1 ^b^ | Ref. | 0.86 (0.75,0.98)* | 0.89 (0.78,1.02) | 0.78 (0.67,0.90)*** | 0.93 (0.88,0.98)** |
| Model 2 ^c^ | Ref. | 0.87 (0.76,0.99)* | 0.91 (0.79,1.04) | 0.80 (0.69,0.93)** | 0.94 (0.89,0.99)* |
| **Spain** |  |  |  |  |  |
| Cases/n | 28/3244 | 61/3217 | 66/2913 | 65/2690 | 220/12064 |
| Model 1 ^b^ | Ref. | 0.97 (0.62,1.52) | 0.87 (0.56,1.35) | 0.81 (0.52,1.26) | 0.93 (0.80,1.08) |
| Model 2 ^c^ | Ref. | 0.96 (0.61,1.51) | 0.91 (0.58,1.41) | 0.87 (0.55,1.36) | 0.96 (0.83,1.12) |
| **Czechia** |  |  |  |  |  |
| Cases/n | 214/2606 | 155/2071 | 147/1910 | 133/1776 | 649/8363 |
| Model 1 ^b^ | Ref. | 0.87 (0.71,1.07) | 0.88 (0.71,1.08) | 0.81 (0.66,1.01) | 0.93 (0.86,1.01) |
| Model 2 ^c^ | Ref. | 0.90 (0.73,1.10) | 0.89 (0.72,1.09) | 0.83 (0.67,1.03) | 0.94 (0.87,1.02) |
| **Poland** |  |  |  |  |  |
| Cases/n | 190/2870 | 152/2638 | 153/2242 | 102/1882 | 597/9632 |
| Model 1 ^b^ | Ref. | 0.79 (0.63,0.97)* | 0.92 (0.74,1.14) | 0.70 (0.55,0.89)** | 0.92 (0.84,0.99)* |
| Model 2 ^c^ | Ref. | 0.78 (0.63,0.97)* | 0.93 (0.75,1.15) | 0.71 (0.56,0.91)** | 0.92 (0.85,1.00)* |
| **UK** |  |  |  |  |  |
| Cases/n | 32/1537 | 33/1428 | 24/1275 | 26/1168 | 115/5408 |
| Model 1 ^b^ | Ref. | 1.06 (0.65,1.72) | 0.88 (0.52,1.49) | 0.95 (0.57,1.60) | 1.00 (0.83,1.20) |
| Model 2 ^c^ | Ref. | 1.08 (0.66,1.76) | 0.96 (0.56,1.63) | 1.07 (0.64,1.80) | 1.05 (0.88,1.26) |

**Table S5** (continued)

|  | **Dietary Approaches to Stop Hypertension ^a^** | | | | |
| --- | --- | --- | --- | --- | --- |
|  | **Quartile 1 (lowest)** | **Quartile 2** | **Quartile 3** | **Quartile 4 (highest)** | **Per 1-SD increment** |
| Cancer mortality |  |  |  |  |  |
| **Pooled sample** |  |  |  |  |  |
| Cases/n | 484/10257 | 490/9354 | 471/8340 | 369/7516 | 1814/35467 |
| Model 1 ^b^ | Ref. | 1.01 (0.89,1.15) | 1.03 (0.91,1.17) | 0.84 (0.73,0.97)* | 0.93 (0.89,0.98)** |
| Model 2 ^c^ | Ref. | 1.06 (0.93,1.20) | 1.11 (0.98,1.27) | 0.94 (0.82,1.08) | 0.97 (0.92,1.02) |
| **Spain** |  |  |  |  |  |
| Cases/n | 41/3244 | 73/3217 | 80/2913 | 83/2690 | 277/12064 |
| Model 1 ^b^ | Ref. | 0.94 (0.64,1.38) | 0.86 (0.59,1.26) | 0.83 (0.57,1.21) | 0.94 (0.82,1.07) |
| Model 2 ^c^ | Ref. | 0.94 (0.64,1.38) | 0.93 (0.63,1.36) | 0.94 (0.64,1.37) | 0.99 (0.86,1.12) |
| **Czechia** |  |  |  |  |  |
| Cases/n | 180/2606 | 180/2071 | 170/1910 | 129/1776 | 659/8363 |
| Model 1 ^b^ | Ref. | 1.19 (0.97,1.46) | 1.19 (0.97,1.47) | 0.95 (0.76,1.19) | 0.98 (0.90,1.06) |
| Model 2 ^c^ | Ref. | 1.27 (1.03,1.56)* | 1.27 (1.03,1.56)* | 1.05 (0.84,1.32) | 1.01 (0.94,1.10) |
| **Poland** |  |  |  |  |  |
| Cases/n | 190/2870 | 185/2638 | 168/2242 | 103/1882 | 646/9632 |
| Model 1 ^b^ | Ref. | 0.97 (0.79,1.18) | 1.02 (0.83,1.26) | 0.71 (0.55,0.90)** | 0.89 (0.82,0.97)** |
| Model 2 ^c^ | Ref. | 1.01 (0.82,1.23) | 1.12 (0.91,1.38) | 0.78 (0.62,1.00)* | 0.93 (0.86,1.01) |
| **UK** |  |  |  |  |  |
| Cases/n | 73/1537 | 52/1428 | 53/1275 | 54/1168 | 232/5408 |
| Model 1 ^b^ | Ref. | 0.72 (0.50,1.02) | 0.82 (0.58,1.17) | 0.87 (0.61,1.24) | 0.91 (0.80,1.03) |
| Model 2 ^c^ | Ref. | 0.75 (0.53,1.07) | 0.91 (0.64,1.30) | 0.98 (0.68,1.39) | 0.95 (0.84,1.09) |

*p<0.05. **p<0.01. ***p<0.001. SD=Standard Deviation.

^a^ Quartiles of the Dietary Approaches to Stop Hypertension: Spain (Quartile 1, 8 to 19; Quartile 2, 20 to 23; Quartile 3, 24 to 27; Quartile 4, 28 to 40), Czechia (Quartile 1, 9 to 21; Quartile 2, 22 to 24; Quartile 3, 25 to 27; Quartile 4, 28 to 39), Poland (Quartile 1, 11 to 21; Quartile 2, 22 to 24; Quartile 3, 25 to 27; Quartile 4, 28 to 39), UK (Quartile 1, 9 to 21; Quartile 2, 22 to 24; Quartile 3, 25 to 27; Quartile 4, 28 to 37).

1-SD increment: Spain, 5.50; Czechia, 4.51; Poland, 4.26; UK, 4.34.

^b^ Model 1: Cox regression model stratified for country (pooled sample), and adjusted for sex, age, educational level (primary or less, secondary or vocational, university, or no data), and marital status (single, married/cohabiting, divorced/separated or widowed, or no data).

^c^ Model 2: As Model 1 and additionally adjusted for tobacco smoking (never, former, current, or no data), leisure-time physical activity (hours/week), energy intake (kcal/day), diabetes, cardiovascular disease history, musculoskeletal disease, chronic lung disease, cancer history and depression.

**Table S6.** Associations between the Southern European Atlantic Diet food groups and 13.6-year mortality in the pooled sample.

|  | All-cause mortality | | Cardiovascular mortality | | Cancer mortality | |
| --- | --- | --- | --- | --- | --- | --- |
|  | **Cases/n** | **HR (95% CI) ^a^** | **Cases/n** | **HR (95% CI) ^a^** | **Cases/n** | **HR (95% CI) ^a^** |
| **Fresh fish (excluding cod)** |  |  |  |  |  |  |
| Less than weekly | 2259/13174 | Ref. | 782/13045 | Ref. | 855/13045 | Ref. |
| 1 to 6 times/week | 2578/21235 | 0.91 (0.84,0.98)* | 761/20931 | 0.95 (0.83,1.08) | 920/20931 | 0.88 (0.78,1.00) |
| Daily | 136/1508 | 0.80 (0.51,1.26) | 38/1491 | 0.57 (0.23,1.43) | 39/1491 | 1.02 (0.53,1.97) |
| **Cod** |  |  |  |  |  |  |
| Less than weekly | 3582/26030 | Ref. | 1154/25728 | Ref. | 1251/25728 | Ref. |
| 1 to 6 times/week | 1382/9856 | 1.02 (0.94,1.10) | 424/9709 | 0.97 (0.84,1.12) | 561/9709 | 1.10 (0.96,1.25) |
| Daily | 9/31 | ^b^ | 3/30 | ^b^ | 2/30 | ^b^ |
| **Red meat and pork products** |  |  |  |  |  |  |
| Less than weekly | 216/1653 | Ref. | 57/1642 | Ref. | 64/1642 | Ref. |
| 1 to 6 times/week | 2613/19220 | 0.93 (0.68,1.26) | 854/19000 | 1.25 (0.70,2.21) | 956/19000 | 0.76 (0.48,1.20) |
| Daily | 2144/15044 | 0.94 (0.69,1.28) | 670/14825 | 1.20 (0.67,2.13) | 794/14825 | 0.76 (0.48,1.20) |
| **Dairy** |  |  |  |  |  |  |
| Less than weekly | 86/498 | Ref. | 25/490 | Ref. | 31/490 | Ref. |
| 1 to 6 times/week | 1116/6775 | 0.97 (0.75,1.25) | 388/6680 | 1.09 (0.71,1.67) | 410/6680 | 0.89 (0.59,1.34) |
| Daily | 3771/28644 | 0.94 (0.73,1.20) | 1168/28297 | 0.98 (0.64,1.50) | 1373/28297 | 0.84 (0.56,1.27) |
| **Legumes and vegetables** |  |  |  |  |  |  |
| Less than weekly | 24/172 | Ref. | 12/172 | Ref. | 5/172 | Ref. |
| 1 to 6 times/week | 445/3579 | 0.90 (0.53,1.51) | 141/3534 | 0.59 (0.29,1.22) | 129/3534 | ^b^ |
| Daily | 4504/32166 | 0.74 (0.44,1.22) | 1428/31761 | 0.45 (0.23,0.90)* | 1680/31761 | ^b^ |
| **Vegetable soup** |  |  |  |  |  |  |
| Less than weekly | 1591/16975 | Ref. | 411/16848 | Ref. | 565/16848 | Ref. |
| 1 to 6 times/week | 3013/17307 | 0.91 (0.82,1.01) | 1024/17004 | 1.01 (0.84,1.21) | 1124/17004 | 0.91 (0.76,1.07) |
| Daily | 369/1635 | 0.99 (0.85,1.14) | 146/1615 | 1.17 (0.92,1.50) | 125/1615 | 0.90 (0.70,1.15) |
| **Potatoes** |  |  |  |  |  |  |
| Less than weekly | 384/4084 | Ref. | 103/4039 | Ref. | 107/4039 | Ref. |
| 1 to 6 times/week | 3707/26334 | 1.24 (0.97,1.58) | 1189/25999 | 1.00 (0.68,1.45) | 1377/25999 | 1.32 (0.88,1.99) |
| Daily | 882/5499 | 1.30 (1.01,1.68)* | 289/5429 | 1.06 (0.72,1.58) | 330/5429 | 1.27 (0.83,1.95) |
| **Whole-grain bread** |  |  |  |  |  |  |
| Less than weekly | 2006/16018 | Ref. | 578/15801 | Ref. | 666/15801 | Ref. |
| 1 to 6 times/week | 789/6082 | 0.94 (0.85,1.04) | 255/6004 | 0.95 (0.80,1.13) | 301/6004 | 0.90 (0.76,1.06) |
| Daily | 2178/13817 | 0.94 (0.87,1.02) | 748/13662 | 0.94 (0.82,1.07) | 847/13662 | 0.98 (0.86,1.12) |
| **Wine** |  |  |  |  |  |  |
| 0 or >1 glass/day (women), 0 or >2 glasses/day (men) | 3456/22513 | Ref. | 1151/22162 | Ref. | 1202/22162 | Ref. |
| ≥0 to 1 glass/day (women), ≥0 to 2 glasses/day (men) | 1517/13404 | 0.81 (0.75,0.88)*** | 430/13305 | 0.70 (0.61,0.81)*** | 612/13305 | 0.83 (0.73,0.95)** |

*p<0.05. **p<0.01. ***p<0.001. CI = confidence interval. HR = hazard ratio

^a^ Cox regression model stratified for country and adjusted for sex, age, educational level (primary or less, secondary or vocational, university, or no data), marital status (single, married/cohabiting, divorced/separated or widowed, or no data), tobacco smoking (never, former, current, or no data), leisure-time physical activity (hours/week), energy intake (kcal/day), diabetes, cardiovascular disease history, musculoskeletal disease, chronic lung disease, cancer history, depression, and all other SEAD food groups.

^b^ <10 cases in the lowest or highest category may render unreliable hazard ratios.

**Table S7.** Sensitivity analyses. Associations between the Southern European Atlantic diet (per 1-standard deviation increment) and 13.6-year mortality.

|  | All-cause mortality | | Cardiovascular mortality | | Cancer mortality | |
| --- | --- | --- | --- | --- | --- | --- |
|  | **Cases/n** | **Model 2 ^a^** | **Cases/n** | **Model 2 ^a^** | **Cases/n** | **Model 2 ^a^** |
| **Pooled sample** |  |  |  |  |  |  |
| SEAD optimised for potential public health interventions ^b^ | 4973/35917 | 0.95 (0.92,0.98)*** | 1581/35467 | 0.97 (0.92,1.02) | 1814/35467 | 0.96 (0.92,1.01) |
| SEAD considering total alcohol intake ^c^ | 4973/35917 | 0.92 (0.89,0.95)*** | 1581/35467 | 0.91 (0.87,0.96)** | 1814/35467 | 0.94 (0.89,0.99)* |
| Adjusting for common foods not included in the SEAD ^d^ | 4973/35917 | 0.92 (0.89,0.95)*** | 1581/35467 | 0.92 (0.87,0.97)** | 1814/35467 | 0.94 (0.89,0.99)* |
| Adjusting for hypertension ^e^ | 4973/35917 | 0.92 (0.89,0.95)*** | 1581/35467 | 0.91 (0.86,0.96)*** | 1814/35467 | 0.94 (0.89,0.99)* |
| Adjusting for body mass index | 4973/35917 | 0.92 (0.90,0.95)*** | 1581/35467 | 0.91 (0.86,0.96)*** | 1814/35467 | 0.94 (0.90,0.99)* |
| Not adjusting for morbidity | 4973/35917 | 0.93 (0.90,0.96)*** | 1581/35467 | 0.93 (0.88,0.98)** | 1814/35467 | 0.94 (0.90,0.99)* |
| Omitting the first year of follow up | 4818/35762 | 0.92 (0.89,0.95)*** | 1524/35320 | 0.91 (0.86,0.96)*** | 1757/35320 | 0.94 (0.89,0.99)* |
| Excluding the subjects with severe chronic diseases | 2256/25292 | 0.89 (0.85,0.93)*** | 591/25067 | 0.91 (0.83,1.00)* | 912/25067 | 0.89 (0.83,0.96)** |
| Estimating relative risks of death at 15 years ^f^ | 4973/35917 | 0.96 (0.93,0.98)*** | 1581/35467 | 0.94 (0.89,1.00) | 1814/35467 | 0.97 (0.91,1.04) |
| **Spain** |  |  |  |  |  |  |
| SEAD optimised for potential public health interventions ^b^ | 974/12191 | 1.03 (0.97,1.10) | 220/12064 | 1.13 (0.99,1.29) | 277/12064 | 0.99 (0.88,1.11) |
| SEAD considering total alcohol intake ^c^ | 974/12191 | 0.94 (0.88,1.00) | 220/12064 | 0.95 (0.83,1.08) | 277/12064 | 0.99 (0.88,1.12) |
| Adjusting for common foods not included in the SEAD ^d^ | 974/12191 | 0.93 (0.88,0.99)* | 220/12064 | 0.95 (0.84,1.09) | 277/12064 | 0.98 (0.87,1.10) |
| Adjusting for hypertension ^e^ | 974/12191 | 0.93 (0.88,0.99)* | 220/12064 | 0.95 (0.83,1.08) | 277/12064 | 0.98 (0.87,1.10) |
| Adjusting for body mass index | 974/12191 | 0.93 (0.88,0.99)* | 220/12064 | 0.95 (0.83,1.08) | 277/12064 | 0.98 (0.87,1.10) |
| Not adjusting for morbidity | 974/12191 | 0.95 (0.89,1.01) | 220/12064 | 0.96 (0.85,1.10) | 277/12064 | 1.00 (0.89,1.12) |
| Omitting the first year of follow up | 960/12177 | 0.94 (0.88,1.00)* | 214/12050 | 0.95 (0.83,1.08) | 274/12050 | 0.98 (0.87,1.10) |
| Excluding the subjects with severe chronic diseases | 582/10437 | 0.92 (0.85,1.00)* | 114/10356 | 0.95 (0.79,1.14) | 184/10356 | 0.93 (0.80,1.07) |
| Estimating relative risks of death at 15 years ^f^ | 974/12191 | 0.96 (0.92,1.01) | 220/12064 | 0.96 (0.82,1.12) | 277/12064 | 1.03 (0.94,1.14) |
| **Czechia** |  |  |  |  |  |  |
| SEAD optimised for potential public health interventions ^b^ | 1696/8372 | 0.96 (0.91,1.01) | 649/8363 | 0.95 (0.88,1.03) | 659/8363 | 0.99 (0.92,1.07) |
| SEAD considering total alcohol intake ^c^ | 1696/8372 | 0.94 (0.89,0.98)* | 649/8363 | 0.94 (0.87,1.02) | 659/8363 | 0.92 (0.85,0.99)* |
| Adjusting for common foods not included in the SEAD ^d^ | 1696/8372 | 0.94 (0.89,0.99)* | 649/8363 | 0.93 (0.86,1.01) | 659/8363 | 0.92 (0.85,0.99)* |
| Adjusting for hypertension ^e^ | 1696/8372 | 0.94 (0.89,0.99)* | 649/8363 | 0.92 (0.85,1.00)* | 659/8363 | 0.92 (0.85,1.00)* |
| Adjusting for body mass index | 1696/8372 | 0.94 (0.90,0.99)* | 649/8363 | 0.92 (0.85,1.00)* | 659/8363 | 0.92 (0.85,1.00)* |
| Not adjusting for morbidity | 1696/8372 | 0.96 (0.91,1.01) | 649/8363 | 0.96 (0.88,1.04) | 659/8363 | 0.93 (0.86,1.00) |
| Omitting the first year of follow up | 1638/8314 | 0.94 (0.89,0.99)* | 629/8309 | 0.92 (0.85,1.00) | 638/8309 | 0.91 (0.84,0.99)* |
| Excluding the subjects with severe chronic diseases | 691/5104 | 0.87 (0.81,0.94)*** | 233/5098 | 0.93 (0.81,1.06) | 303/5098 | 0.86 (0.77,0.97)* |
| Estimating relative risks of death at 15 years ^f^ | 1696/8372 | 0.98 (0.93,1.02) | 649/8363 | 0.97 (0.89,1.06) | 659/8363 | 0.93 (0.83,1.05) |

**Table S7.** (continued)

|  | All-cause mortality | | Cardiovascular mortality | | Cancer mortality | |
| --- | --- | --- | --- | --- | --- | --- |
|  | **Cases/n** | **Model 2 ^b^** | **Cases/n** | **Model 2 ^b^** | **Cases/n** | **Model 2 ^b^** |
| **Poland** |  |  |  |  |  |  |
| SEAD optimised for potential public health interventions ^b^ | 1839/9944 | 0.89 (0.85,0.94)*** | 597/9632 | 0.90 (0.83,0.97)** | 646/9632 | 0.92 (0.85,0.99)* |
| SEAD considering total alcohol intake ^c^ | 1839/9944 | 0.89 (0.85,0.93)*** | 597/9632 | 0.85 (0.79,0.92)*** | 646/9632 | 0.93 (0.86,1.00) |
| Adjusting for common foods not included in the SEAD ^d^ | 1839/9944 | 0.89 (0.85,0.93)*** | 597/9632 | 0.86 (0.79,0.93)*** | 646/9632 | 0.92 (0.85,1.00)* |
| Adjusting for hypertension ^e^ | 1839/9944 | 0.89 (0.85,0.93)*** | 597/9632 | 0.86 (0.79,0.93)*** | 646/9632 | 0.92 (0.85,0.99)* |
| Adjusting for body mass index | 1839/9944 | 0.89 (0.85,0.93)*** | 597/9632 | 0.86 (0.79,0.93)*** | 646/9632 | 0.92 (0.85,1.00)* |
| Not adjusting for morbidity | 1839/9944 | 0.89 (0.85,0.93)*** | 597/9632 | 0.87 (0.80,0.94)*** | 646/9632 | 0.91 (0.84,0.98)* |
| Omitting the first year of follow up | 1765/9870 | 0.89 (0.85,0.93)*** | 569/9562 | 0.85 (0.79,0.93)*** | 617/9562 | 0.93 (0.86,1.01) |
| Excluding the subjects with severe chronic diseases | 726/5918 | 0.88 (0.82,0.95)*** | 192/5781 | 0.84 (0.73,0.97)* | 280/5781 | 0.91 (0.80,1.02) |
| Estimating relative risks of death at 15 years ^f^ | 1839/9944 | 0.93 (0.90,0.96)*** | 597/9632 | 0.88 (0.81,0.96)** | 646/9632 | 0.96 (0.89,1.04) |
| **UK** |  |  |  |  |  |  |
| SEAD optimised for potential public health interventions ^b^ | 464/5410 | 0.98 (0.89,1.07) | 115/5408 | 1.11 (0.92,1.33) | 232/5408 | 0.97 (0.86,1.11) |
| SEAD considering total alcohol intake ^c^ | 464/5410 | 0.97 (0.89,1.06) | 115/5408 | 1.05 (0.88,1.26) | 232/5408 | 0.99 (0.87,1.13) |
| Adjusting for common foods not included in the SEAD ^d^ | 464/5410 | 0.98 (0.89,1.07) | 115/5408 | 1.04 (0.87,1.24) | 232/5408 | 1.02 (0.89,1.16) |
| Adjusting for hypertension ^e^ | 464/5410 | 0.98 (0.89,1.07) | 115/5408 | 1.03 (0.86,1.23) | 232/5408 | 1.02 (0.89,1.16) |
| Adjusting for body mass index | 464/5410 | 0.98 (0.89,1.07) | 115/5408 | 1.03 (0.86,1.23) | 232/5408 | 1.02 (0.90,1.17) |
| Not adjusting for morbidity | 464/5410 | 0.98 (0.90,1.07) | 115/5408 | 1.04 (0.87,1.25) | 232/5408 | 1.03 (0.90,1.17) |
| Omitting the first year of follow up | 455/5401 | 0.97 (0.89,1.06) | 112/5399 | 1.01 (0.85,1.21) | 228/5399 | 1.01 (0.88,1.15) |
| Excluding the subjects with severe chronic diseases | 257/3833 | 0.87 (0.78,0.99)* | 52/3832 | 1.03 (0.78,1.36) | 145/3832 | 0.88 (0.75,1.03) |
| Estimating relative risks of death at 15 years ^f^ | 464/5410 | 0.98 (0.88,1.09) | 115/5408 | 1.15 (0.79,1.69) | 232/5408 | 1.13 (0.92,1.40) |

*p<0.05. **p<0.01. ***p<0.001.

Associations are summarised with hazard ratios (95% confidence interval), unless otherwise indicated.

^a^ Model 2: Cox regression model stratified for country (pooled sample) and adjusted for sex, age, educational level (primary or less, secondary or vocational, university, or no data), marital status (single, married/cohabiting, divorced/separated or widowed, or no data), tobacco smoking (never, former, current, or no data), leisure-time physical activity (hours/week), energy intake (kcal/day), diabetes, cardiovascular disease history, musculoskeletal disease, chronic lung disease, cancer history and depression.

^b^ Reverse scoring the consumption of red meat/pork products and potatoes, and not scoring wine consumption. Adjusting for wine consumption.

^c^ Men who had >0 and ≤20 g/day of alcohol, and women who had >0 and ≤10 g/day of alcohol were given 1 point, whereas no points were given for >20 g/day in men, >10 g/day in women, or 0 g/day.

^d^ Fruits, nuts, and sugar-sweetened beverages.

^e^ Systolic blood pressure ≥130 millimetres of mercury, diastolic blood pressure ≥80 millimetres of mercury, or use of antihypertensive medication.

^f^ Generalised linear model adjusted for country (pooled sample), sex, age, educational level (primary or less, secondary or vocational, university, or no data), marital status (single, married/cohabiting, divorced/separated or widowed, or no data), tobacco smoking (never, former, current, or no data), leisure-time physical activity (hours/week), energy intake (kcal/day), diabetes, cardiovascular disease history, musculoskeletal disease, chronic lung disease, cancer history and depression.

**Figure S1.** Participants’ flow chart.

POOLED SAMPLE (total=40,560)

Participants in the ENRICA study (n=13,105)

Participants in the HAPIEE study (n=19,585)

Participants in the Whitehall II study at phase 5 (n=7,870)

EXCLUDED (total=4,643) ^a^

No data on diet (total=3,578)

- No diet history or FFQ data (n=3,373)
- Implausible energy intake ^b^ (n=205)

No data on mortality (total=1,091)

- Declined mortality follow-up or linkage with mortality register not available (n=1,091)

**Pooled sample for all-cause mortality analyses**

**n=35,917**

EXCLUDED (total=450)

No data on mortality causes (total=450)

**Pooled sample for cause-specific mortality analyses**

**n=35,467**

FFQ = Food Frequency Questionnaire.

^a^ Note that one individual may lack data in more than one variable.

^b^ <500 kcal/day or >5000 kcal/day.

**Figure S2.** Hazard Ratios (95% confidence interval) for the association between the Southern European Atlantic Diet and 13.6-year all-cause mortality, by country.

**
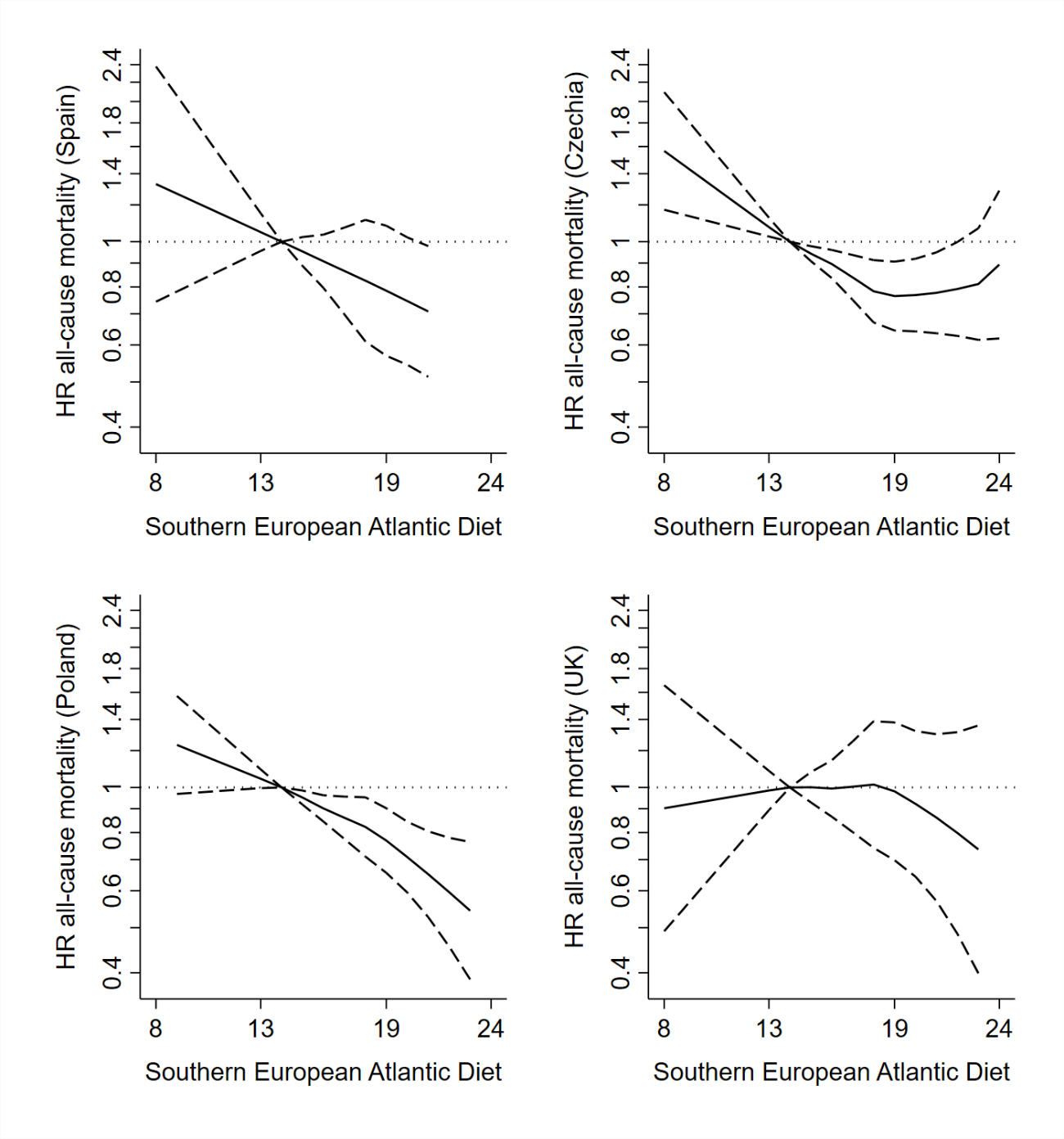
**

Cox regression model adjusted as Model 2 in Table 2: sex, age, educational level (primary or less, secondary or vocational, university, or no data), marital status (single, married/cohabiting, divorced/separated or widowed, or no data), tobacco smoking (never, former, current, or no data), leisure-time physical activity (hours/week), energy intake (kcal/day), diabetes, cardiovascular disease history, musculoskeletal disease, chronic lung disease, cancer history and depression.
